# Synthesis of Tetrasubstituted Nitroalkenes and Preliminary Studies of Their Enantioselective Organocatalytic Reduction

**DOI:** 10.3390/molecules28073156

**Published:** 2023-04-01

**Authors:** Patricia Camarero González, Sergio Rossi, Miguel Sanz, Francesca Vasile, Maurizio Benaglia

**Affiliations:** 1 Dipartimento di Chimica, Università degli Studi di Milano, Via Golgi 19, 20133 Milano, Italy; 2 Taros Chemicals GmbH & Co. KG, Emil-Figge-Str. 76a, 44227 Dortmund, Germany

**Keywords:** nitroacrylates, organocatalysis, stereoselective synthesis, reduction, chiral nitro derivatives

## Abstract

Starting from commercially available ketones, a reproducible and reliable strategy for the synthesis of tetrasubstituted nitroalkenes was successfully developed, using a two-step procedure; the HWE olefination of the ketone to form the corresponding α,β-unsaturated esters is followed by a nitration reaction to introduce the nitro group in the α position of the ester group. The enantioselective organocatalytic reduction of these compounds has also been preliminarily studied, to access the functionalized enantioenriched nitroalkanes, which are useful starting materials for further synthetic elaborations. The absolute configuration of the reduction product was established by chemical correlation of the chiral nitroalkane with a known product; preliminary DFT calculations were also conducted to rationalize the stereochemical outcome of the organocatalytic enantioselective reduction.

## 1. Introduction

Among unnatural α-amino acids, α,α-disubstituted amino acids are key biological scaffolds with many specific roles and properties that have made them increasingly attractive in the fields of organic chemistry, biochemical research and drug discovery [1,2,3]. They have unique structural properties, which make them ideal candidates to be included in the design of new pharmaceutically active compounds, as well as intermediates for the study of pathological pathways [4,5,6].

**Figure 1:**
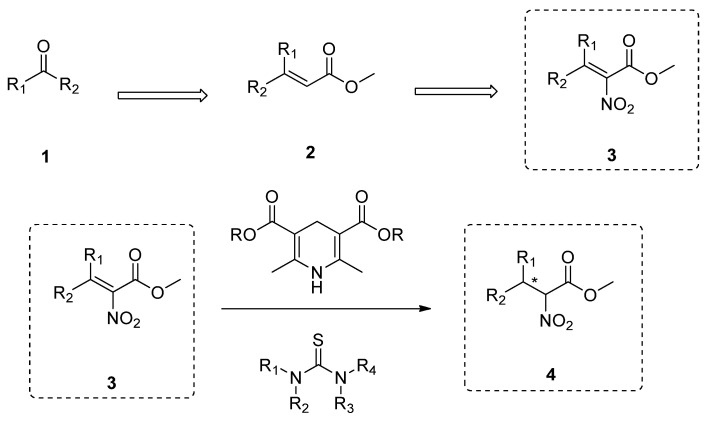
Synthetic approach for the synthesis of tetrasubstituted nitroalkenes and their enantioselective catalytic reduction.

Synthetic approaches for the synthesis of di- or trisubstituted nitroalkenes, valuable intermediates for the synthesis of α,α-disubstituted amino acids, are abundant in the literature, but there are very scarce data reporting synthetic routes for tetrasubstituted nitroalkenes [7,8]. Herein we describe a reproducible methodology for the synthesis of these compounds (Figure 1) that started from easily available ketones (**1**), which are converted to tetrasubstituted nitroolefins (**3**), by reaction of an acrylate intermediate (**2**), with an appropriate nitration reagent. Nitroalkenes will be organocatalytically reduced to afford enantioenriched nitroalkanes (**4**), highly functionalized chiral starting materials for further transformations (Figure 1).

## 2. Results and Discussion

### 2.1. Synthesis of Tetrasubstituted Nitroalkenes

**Scheme 1:**
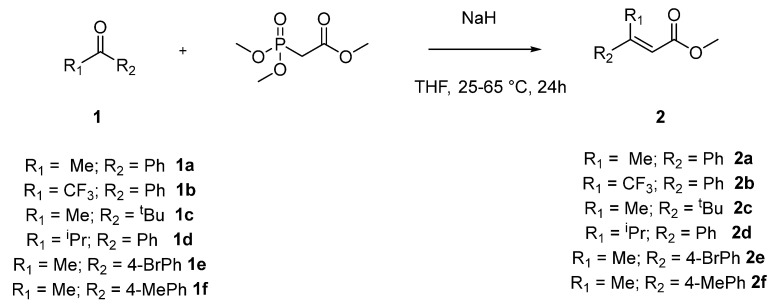
Synthesis of acrylates intermediates (**2**).

We started the investigations by exploring a two-step synthetic strategy for the synthesis of tetrasubstituted nitroalkenes (Scheme 1). The first reaction involves the formation of acrylates **2a**–**f**, by reaction of different commercially available ketones **1a**–**f**, using Horner–Wadstworth–Emmons reaction conditions [9]. The results are summarized in Table 1.

Compounds **2a**–**f** were obtained after reaction of the corresponding commercially available ketones with trimethylphosphonoacetate and sodium hydride in THF for 24 h with good to moderate yields and excellent diastereoselectivities after purification with column chromatography. The reactions were performed at room temperature, but with compounds **2c**, **2d** and **2f** (Table 1, entries 3,4,6) it was necessary to heat the reaction to 66 °C. Their E/Z ratio was checked by ^1^H-NMR of reaction crude. The low yield observed for the isolation of pure product **2c** (Table 1, entry 3) can be explained by its high volatility, and optimization of the product isolation is underway.

Then, our efforts were concentrated on the nitration reaction of these acrylate intermediates using common nitration reagents such as HNO_3_ [10,11]. However, the use of nitric acid mainly led to the formation of products with the nitro group on the aromatic ring, and the yields after purification were very low (<10%). Furthermore, several problems were also detected during the isolation process. Selective nitration conditions for double bonds were also considered, but no formation of the desired nitroalkane was observed. Thus, alternative strategies involving the condensation between acetophenone and ethyl nitroacetate or the reaction between phenylacetylene with ethyl nitroacetate catalyzed by indium salts were also explored, [12,13,14,15] but without any satisfactory results (see Supplementary Materials).

**Scheme 2:**
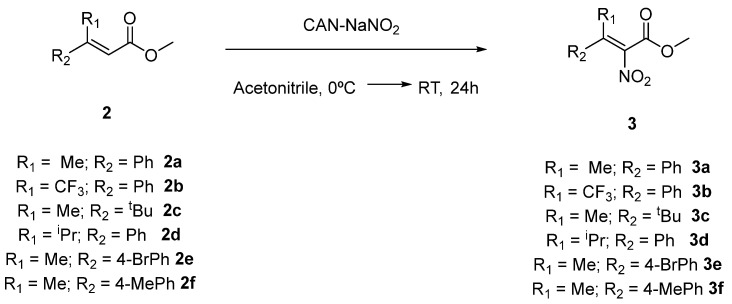
Synthesis of tetrasubstituted nitroalkenes **3a**–**f**.

Finally, Buevich and co-workers reported the first example of a α-nitro addition to a cinammic ester for the synthesis of dehydrophenylalanine derivatives, which are precursors of α-amino acids, by utilizing a CAN-NaNO_2_ system [16]. Therefore, we decided to investigate the methodology for the synthesis of target tetrasubstituted nitroalkenes **3a**–**f** starting from acrylates **2a**–**f** (Scheme 2).

The results are summarized in Table 2.

The nitration reaction of acrylates led to the formation of the corresponding tetrasubstituted nitroacrylates **3a**–**f** in low to moderate yields after chromatographic purification, and typically in a 6/4 diasteoisomeric ratio, except in the case of **3d,** when a single isomer was isolated. In case of nitroacrylates **3a**,**e** (Table 2, entries 1 and 5), two different fractions corresponding to the two separated diastereoisomers were obtained after purification., while for product **3f** (Table 2, entry 6) a single fraction containing both, non-separable, isomers, was obtained after chromatography.

The nitration of acrylate **2b** (Table 2, entry 2), did not lead to the formation of the corresponding nitroacrylate **3b**, but (Z)-methyl 4,4,4-trifluoro-3-(4-nitrophenyl)but-2-enoate was obtained as major compound in this reaction in 76% yield. The nitration of acrylate **2c** (Table 2, entry 3) did not afford any product.

**Figure 2:**
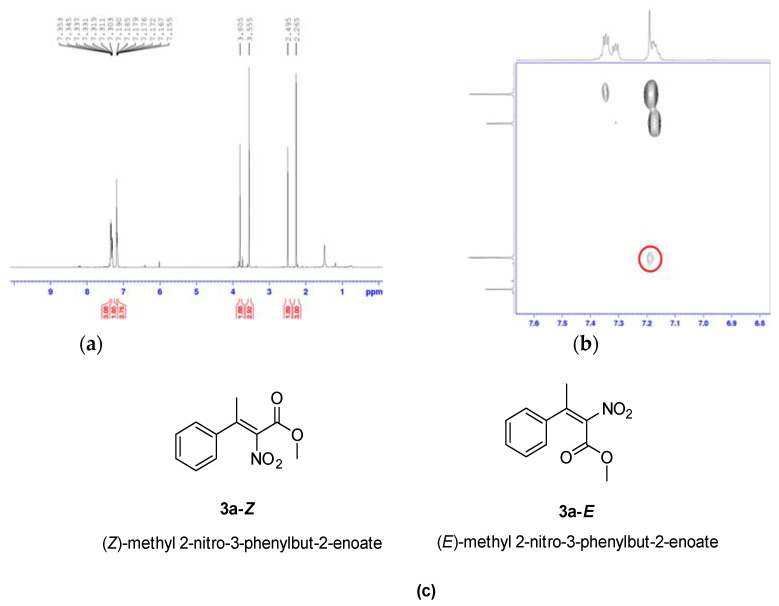
Further NMR experiments performed for tetrasubstituted nitroacrylate **3a.** (**a**): NMR spectra of a mixture fraction of compound **3a**. (**b**): NOE contact observed between OMe and Ph groups in the more abundant form of the tetrasubstituted nitroacrylate **3a**. (**c**): structures of nitroacrylates **3a–*Z*** and **3a–*E***.

As previously mentioned, in the nitration of acrylates **2a**,**e** (Table 2, entries 1 and 5), it was possible to separate the two diastereoisomers. In order to clarify the configuration of the two products, additional NMR experiments were conducted on the isomers of compound **3a** (Figure 2).

A chemical shift study on the methoxy group signal of both Z/E forms of the tetrasubstituted nitroalkene **3a** has been performed, using a NOESY experiment. As illustrated in Figure 2a, the OMe group of the molecule is more shielded (3.55 ppm) in the more abundant form, suggesting that it can be near to the shielding cone of the aromatic ring (corresponding to the (*E*) isomer), while it resonates at 3.8 ppm in the less abundant isomer of Figure 2a.

NOESY experimental results, showing through-space correlation within the molecule, were also acquired to predict if the more abundant isomer of the synthetized tetrasubstituted nitroalkene corresponds to (*E*) or (*Z*), considering that the NOE contact between the methoxy group and the phenyl ring can only be observed in the (*E*) isomer. The analysis of NOE contacts suggested that the significant cross peak between the OMe group and phenyl ring (Figure 2b) is present only in the major isomer that can be determined to have the (*E*) configuration (product **3a**–***E***).

### 2.2. Enantioselective Reduction of Tetrasubstituted Nitroalkenes (***3***)

The use of Hantzsch esters as biomimetic reducing agents [17] has been reported in different organocatalytic reductions of nitroalkenes, but also ketoimines and ketoesters [18,19,20]. In addition, Hantzsch esters are easily synthetized, and their structure readily tuned in order to maximize the efficiency of enantioselective reactions [21].

**Scheme 3:**
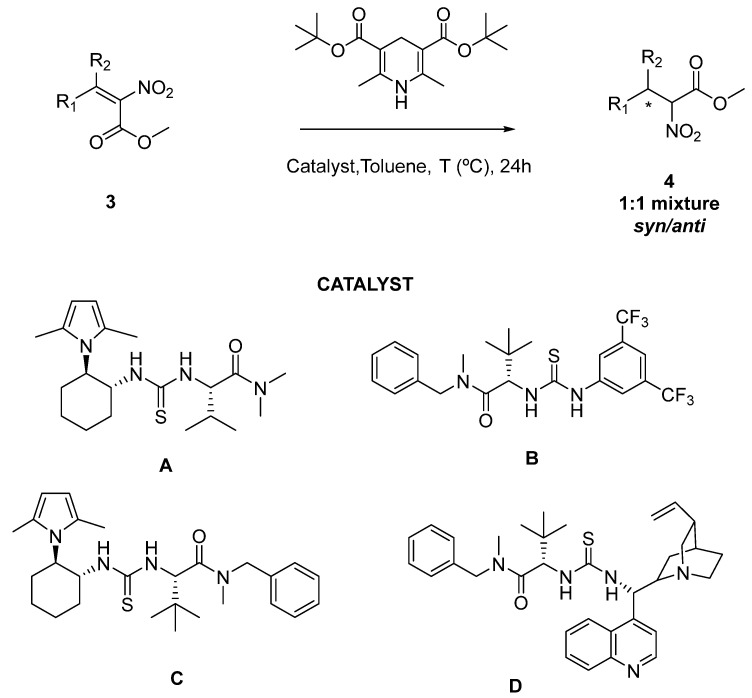
Organocatalytic reduction of tetrasubstituted nitroalkenes.

The enantioselective reduction of the synthetized tetrasubstituted nitroalkenes (**3**) using Hantzsch esters as a reductive agent and a thiourea based chiral catalyst was investigated to obtain the corresponding functionalized nitroalkanes (**4**) (Scheme 3), in the presence of a few chiral bifunctional catalysts **A**–**D**, representative of different classes of the most popular organocatalysts for this transformation. The results are reported in Table 3.

In general, compounds **4** were obtained in a 1:1 mixture of *syn/anti* products after 24 h of reaction time. Initial experiments were conducted using nitroalkane **3a** (Table 3, entries 1–8) as model substrate. The reactions performed in the presence of catalyst **A** starting from different mixtures of nitroalkene **3a** (Table 3, entries 1–3), demonstrate a different reactivity of the *E*–*Z* isomers of this compound, showing that one isomer reacted more quickly than the other one. This interesting discovery was confirmed when the reaction was performed with a pure fraction of the less reactive isomer (which was previously assigned as having a *Z* configuration) and no reaction was observed (Table 3, entry 1). Starting from differently enriched mixtures in the *E* isomer led to similar results (entries 2–3), leading to the chiral alkanes in up to 67% enantiomeric excess (entries 2–3). Attempts to increase the yield by operating at a higher temperature or to improve the enantioselectivity by running the reaction at a lower temperature did not lead to any significant results. The enantioselectivity of the reaction was measured by HPLC analysis of the pure samples on the chiral stationary phase, and two pairs of enantiomers, corresponding to *syn/anti* products, were found (see experimental section).

When thiourea catalyst **B** was used (Table 3, entry 6), the corresponding nitroalkane **3a** was obtained with good yield, but the enantioselectivity observed was lower than with catalyst **A** (Table 3, entry 2), which was the best catalyst for this transformation. When the thiourea catalysts **C** and **D** were tested (Table 3, entries 7,8), the yields were drastically reduced, with a very low quantity of product obtained after purification. With the optimized conditions in hand, the enantioselective reduction of nitroalkenes **3d**–**f** (Table 3, entries 9–11) was carried out using Hanztsch ester and thiourea catalyst **A**. Compounds **4d**–**f** were obtained with good yields after purification, but generally low or modest enantioselectivities.

### 2.3. Determination of the Absolute Configuration of Nitroalkanes (***4***)

**Scheme 4:**
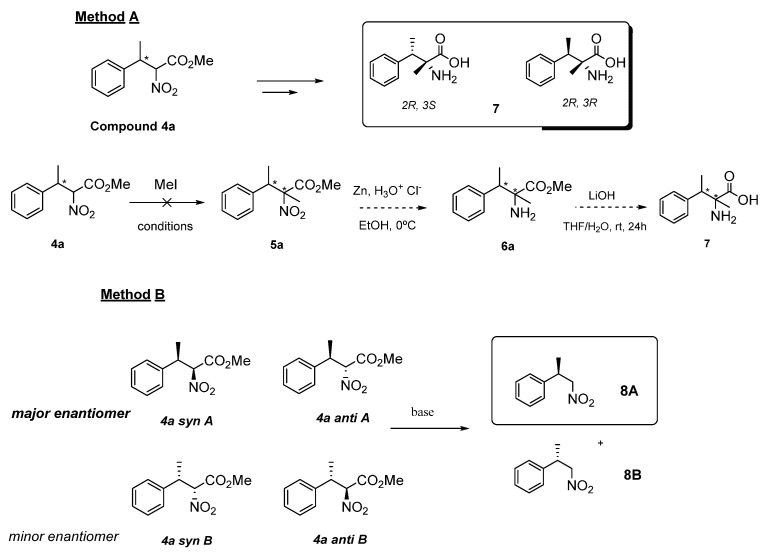
Strategies for the experimental determination of the absolute configuration of nitroal-kane **4a**.

The absolute configuration of the reduction product, the nitroalkane (**4**), was experimentally established by converting **4a** into a known compound. To achieve this goal, two approaches were attempted (Scheme 4).

The first strategy (Scheme 4, method A) explored the transformation of **4a** into the known compound **7**, by a three-step procedure involving the alkylation of nitroalkane **4a**, followed by the reduction of the nitro group and hydrolysis of the ester moiety as depicted in Scheme 4 [22,23,24]. However, despite several conditions being attempted, the alkylation did not lead to the desired product, but mixtures of unreacted starting material and O-alkylated products were obtained (In an additional experimental effort, we have observed that nitroalkane **4a** reacted with “softer” electrophiles, such as methyl vinyl ketone in a Michael reaction, additional studies are underway to further explore these transformations).

Thus, our efforts were focused on an alternative approach (Scheme 4, Method B). The decarboxylation of the ester moiety of **4a** (as 50:50 mixture of *syn/anti* isomers, enantioenriched) to afford the corresponding trisubstituted nitroalkane **8** was then explored [25]. This simple approach was found to be effective and the desired decarboxylated nitroalkane **8** was finally synthetized. The experimental optical rotation was measured using a polarimeter and by comparison with the literature data, the compound **8**, derived from the major enantiomer of product **4a** (***4a syn A*** and ***4a anti A***)**,** was established to have the *R*-configuration [26].

**Figure 3:**
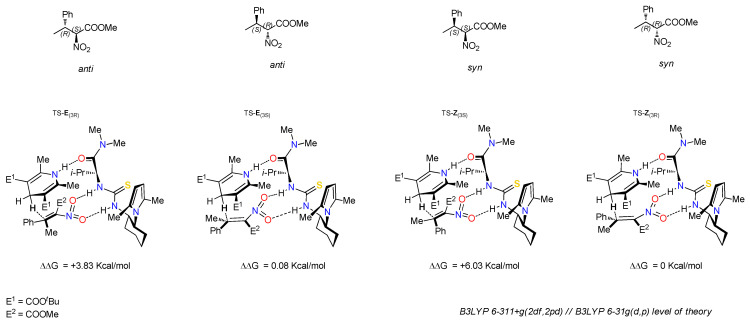
The four possible TS geometries for nitroalkene **3a** complexes with catalyst **A**.

DFT computational studies on the enantioselective reduction of tetrasubstituted nitroalkanes were preliminarily performed to rationalize the stereochemical outcome of the reaction, using the Gaussian g16 package using Catalyst **A** as the model catalyst. All geometries of reactants and products (ground states and transition states) were located at a B3LYP/6-31G (d,p) level of theory and finer electronic energies were successively obtained, increasing the basis set up to 6/311 + (2df,2pd) with B3LYP functional [27]. In Figure 3 four possible complexes of nitroalkene **3a** with catalyst **A** and the geometries of the TS leading to the formation of the four stereoisomers of nitroalkane **4a** are represented.

**Figure 4:**
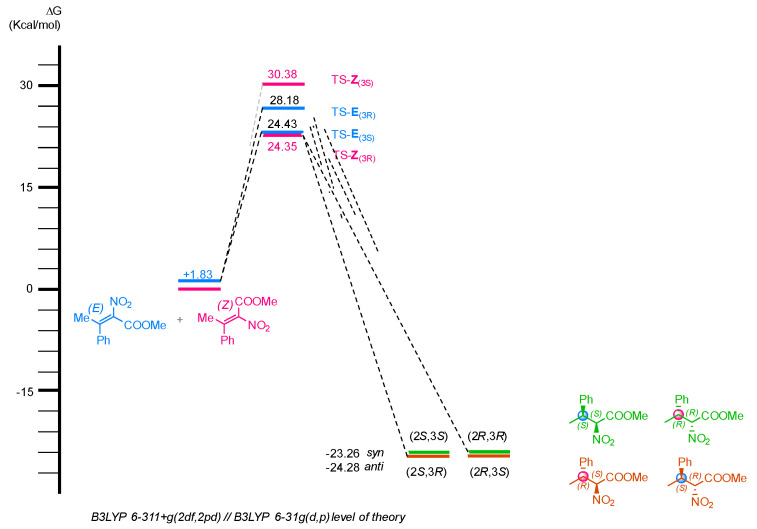
DFT calculations performed for the enantioselective reduction of tetrasubstituted nitroal-kene **3a**.

Transition states responsible for the hydride transfer were located assuming the coordination of the nitro group of the nitroalkane **3a** to the thiourea moiety and of the Hantzsch ester NH group with the catalyst carboxyamide group, according to the so-called Takemoto model. The energy profile is depicted in Figure 4.

**Figure 5:**
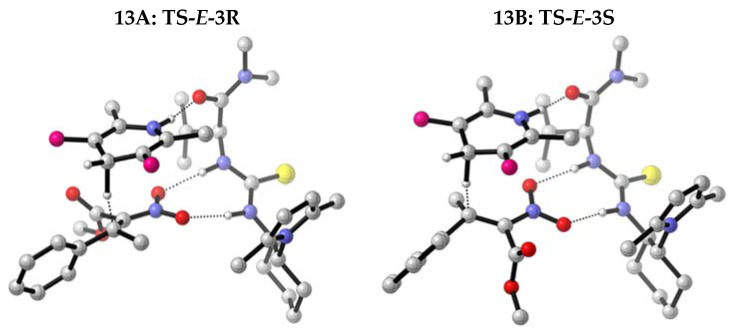
Transition states formed by the *E*- isomer of nitroacrylate **3a**.

In Figure 5 the geometries of the transition states originated by the *E*-isomer of compound **3a** are illustrated.

The blue spheres represent the nitrogen atom of the nitro group of nitroalkane **3a** and of the thiourea catalyst **A**, the yellow one represents the sulfur atom of the thiourea moiety, and the red spheres represent the oxygen atoms of nitro and ester groups of compound **3a,** and of the carboxyamide group of the catalyst. The pink sphere represents the *tert*-butyl groups of the Hantzsch ester, carbon atoms are grey and hydrogen atoms are white. The broken lines showed the H-bonding interaction between the nitro group of **3a** and the thiourea moiety of the catalyst, and the transfer hydride between Hantzsch ester and carbon C_3_ of the nitroolefin.

According to the calculations, among the two transition states originating from *Z*-olefin, TS-*Z*-(*3R*) is the lowest in energy and would lead to the formation of the final product with a *R-*configuration at the C_3_ carbon, in agreement with the experimental data. However, the (*Z*) isomer was found to be very poorly reactive, while, as established in NMR analysis, the more reactive isomer is the (*E*)-nitroacrylate, that, according to the calculations should preferably afford the (S) enantiomer at C_3_ carbon of compound **4a**. These findings are in contrast with the experimentally established absolute configuration (*R*) for the major enantiomer derived from the reaction of the E isomer of **3a**. Therefore, we can conclude that, at the moment, the proposed TS according to the Takemoto model, is not able to explain why the *E* isomer should be more reactive than the *Z* isomer, and, furthermore, cannot predict the correct configuration at the C_3_ of the nitroalkane. Those results are probably an indication that other coordination modes are active in the TS of the reactions, and other models need to be taken into consideration to rationalize the stereochemical outcome of the reaction.

## 3. Materials and Methods

Reactions were monitored by analytical thin-layer chromatography (TLC) using silica gel 60 F_254_ pre-coated glass plates (0.25 mm thickness) and visualized using UV light. Flash chromatography was carried out on silica gel (230–400 mesh). Proton NMR spectra were recorded on spectrometers operating at 300 MHz (Bruker Fourier 300); proton chemical shifts are reported in ppm (δ) with the solvent reference relative to tetramethylsilane (TMS) employed as the internal standard (CDCl_3_: δ = 7.26 ppm). ^13^C-NMR spectra were recorded on 300 MHz spectrometers (Bruker Fourier 300) operating at 75 MHz, with complete proton decoupling; carbon chemical shifts are reported in ppm (δ) relative to TMS with the respective solvent resonance as the internal standard (CDCl_3_: δ = 77.0 ppm). Mass spectra and accurate mass analysis were carried out on a VG AUTOSPEC- M246 spectrometer (double-focusing magnetic sector instrument with EBE geometry) equipped with EI source or with LCQ Fleet ion trap mass spectrometer, ESI source, with acquisition in positive ionization mode in the mass range of 50–2000 m/z. Dry solvents were purchased and stored under nitrogen over molecular sieves (bottles with crown caps). All chemicals were purchased from commercial suppliers and used without further purification unless otherwise specified.

### 3.1. General Procedure for the Synthesis of Tetrasubstituted Nitroalkenes (***3***)

Tetrasubstituted nitroalkenes (**3**) were synthetized using a two-step procedure: Firstly the formation of an acrylate intermediate (**2**) by a Horner–Wasdforth–Emmons reaction of an appropriate ketone (**1**) with trimethylphosphonoacetate and sodium hydride, following by a nitration reaction of this intermediate with a mixture of CAN-NaNO_2_ as an effective nitration reagent.

Compounds **2a**–**f** were synthetized using conditions reported in the literature [9]. First, a solution of trimethyl phosphonoacetate (5.21 mmol) in 20 mL of THF was cooled to 0 °C. Then, sodium hydride (5.21 mmol) was added portion-wise and the mixture was stirred for 30 min. After this time, the appropriate ketone (4.17 mmol) was added at the same temperature and the reaction mixture was allowed to warm to room temperature and stirred for 24 h at the right temperature. Then, 20 mL of saturated solution of ammonium chloride was added dropwise and the mixture was extracted with Et2O. 

The combined organic phases were dried using MgSO_4_, filtered and concentrated in vacuo. The solvent was eliminated under reduced pressure and the crude product was purified using column chromatography and hexanes/EtOAc as eluent. The ^1^H-NMR of compounds **2a**–**f** were in agreement with the published ones. Compounds **2a**–**f** were directly used in the next step after purification.

Acrylates **2a, 2b, 2c, 2d, 2e** and **2f** (5.68 mmol) were dissolved in 50 mL of acetonitrile and cooled to 0 °C. Then, sodium nitrite (17 mmol) and cerium ammonium nitrate (17 mmol) were added at the same temperature, and the reaction mixture was allowed to warm to room temperature and stirred for 24 h. After this time, the reaction was filtered through a pad of celite, and the filtrate was concentrated under reduced pressure. The residue was poured into cold water and extracted with DCM (3 × 50 mL). The combined organic layers were dried using MgSO_4_, filtered and concentrated in vacuo. The crude product was purified by column chromatography using an appropriate mixture of solvents to afford nitroacrylates. For further details see the Supporting Information.

### 3.2. General Procedure for the Enantioselective Synthesis of Nitroalkanes (***4***)

To a stirred solution of nitroalkenes (**3**) in toluene (0.3 mmol 0.3M), catalyst A (10 mol%) and Hanztsch ester (1.2 eq, 0.36 mmol) were added. The reaction mixture was heated at 60 °C for 24 h. Then, the mixture was allowed to warm to room temperature and the solvent was eliminated under reduced pressure, and the crude product was purified using column chromatography and an appropriate mixture of eluents.

## 4. Conclusions

Although the preparation of tetrasubstituted nitroacrylates proved to be very challenging, in this work a reproducible strategy for the synthesis of tetrasubstituted nitroalkenes was successfully developed using a two-step procedure; the HWE olefination of the ketone followed by the reaction of nitration affords the desired tetrasubstituted nitroalkenes (**3**).

The enantioselective reduction of these synthetized tetrasubstituted nitroalkenes (**3**) to access the functionalized nitroalkanes (**4**) was also performed, using a Hantzsch ester as the reductive agent and a thiourea based chiral catalyst, to afford the products with good to moderate yields, in a 1:1 mixture of *syn/anti* isomers, and up to 67% e.e. Although the level of enantioselectivity could not be considered satisfactory yet, it should be noted that the enantioselective organocatalytic reduction of tetrasubsituted alkenes was almost completely unknown. Even if the poor reactivity of the substrates represents a major problem, the present work demonstrates that the asymmetric catalytic reduction of functionalized nitroacrylates may offer a viable strategy for the synthesis of chiral amino ester derivatives.

The absolute configuration of the major enantiomer obtained in the enantioselective reduction was established by converting the nitroalkane **4a** into a known product. DFT calculations, performed in order to rationalize the stereochemical outcome of the reaction did not lead to satisfactory results. Further studies, considering other alternative coordination modes between the catalyst and the substrate, will be necessary in order to understand the origins of the stereocontrol of the reaction.

## Figures and Tables

**Table 1:** Synthesis of acrylate intermediates **2a**–**f**.

Entry	R_1_	R_2_	T	Product	E/Z Ratio ^a^	Yield ^b^
**1**	Me	Ph	RT	**2a**	80:20	52%
**2**	CF_3_	Ph	RT	**2b**	75:25	79%
**3**	Me	^t^Bu	66 °C	**2c**	90:10	14%
**4**	^i^Pr	Ph	66 °C	**2d**	67:33	33%
**5**	Me	4-BrPh	RT	**2e**	65:35	76%
**6**	Me	4-MePh	66 °C	**2f**	75:25	86%

^a^ Determined by ^1^H-NMR. ^b^ Yield was determined after purification using column chromatography.

**Table 2:** Synthesis of tetrasubstituted nitroalkenes **3a**–**f**.

Entry	R_1_	R_2_	Acrylate	Nitroacrylate	Yield ^a^	Isomers Ratio
**1**	Me	Ph	**2a**	**3a**	46%	60/40
**2**	CF_3_	Ph	**2b**	**-**	^b^	-
**3**	Me	^t^Bu	**2c**	**3c**	--- ^c^	-
**4**	^i^Pr	Ph	**2d**	**3d**	25%	99/1
**5**	Me	4-BrPh	**2e**	**3e**	47%	62/38
**6**	Me	4-MePh	**2f**	**3f**	48%	57/43

^a^ Isolated yield after purification with column chromatography. ^b^ Side-product (Z)-methyl 4,4,4-trifluoro-3-(4-nitrophenyl)but-2-enoate was obtained in 76% yield. ^c^ Reaction did not occur, unreacted starting material was recovered.

**Table 3:** Enantioselective reduction of tetrasubstituted nitroalkenes.

Entry	T ^a^	Nitroalkene	Nitroalkane	Catalyst	Yield ^d^ 4	ee% Syn–ee% Anti ^e^
**1** ^a^	60 °C	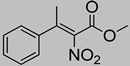 **3a**–***Z***	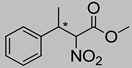 **4a**	**A**	trace	n.d.
**2** ^b^	60 °C	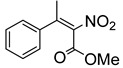 **3a**–***E***	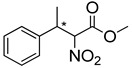 **4a**	**A**	51%	67-63
**3** ^c^	60 °C	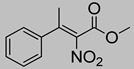 **3a (*E*+*Z*)**	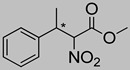 **4a**	**A**	53%	63–52
**4** ^c^	100 °C	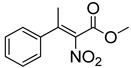 **3a (*E*+*Z*)**	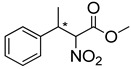 **4a**	**A**	37%	27–22
**5** ^c^	25 °C	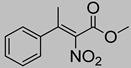 **3a (*E*+*Z*)**	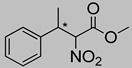 **4a**	**A**	15%	n.d.
**6** ^c^	60 °C	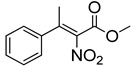 **3a (*E*+*Z*)**	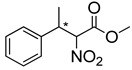 **4a**	**B**	69%	33–32
**7**	60 °C	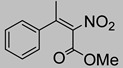 **3a (*E*+*Z*)**	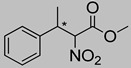 **4a**	**C**	19%	n.d.
**8**	60 °C	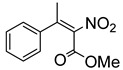 **3a (*E*+*Z*)**	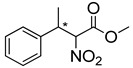 **4a**	**D**	10%	n.d
**9**	60 °C	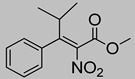 **3d**	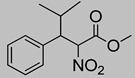 **4d**	**A**	32%	12–13
**10**	60 °C	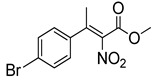 **3e**	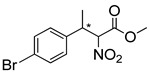 **4e**	**A**	60%	24–4
**11**	60 °C	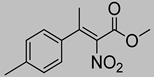 **3f**	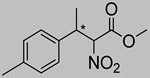 **4f**	**A**	51%	50–46

^a^ Reaction was performed using a 4:96 *E*–*Z* mixture of the nitroalkene; ^b^ Reaction was performed using an 90:10 *E*–*Z* mixture of the nitroalkene; ^c^ Reaction was performed using a 70:30 *E*–*Z* mixture of the nitroalkene; ^d^ Yield was determined after purification with column chromatography; ^e^ Determined using chiral HPLC column Phenomenex-Lux-Cellulose 5, Hexane/IPA 98:2 for compound **4a** and Hexane/IPA 95:5 for compounds **4d**–**f**; n.d. = not determined.

## Data Availability

Data available on request from the authors.

## Supplementary Materials

The following supporting information can be downloaded at: https://www.mdpi.com/article/10.3390/molecules28073156/s1.
