# Supplementary material for: Synthesis of Tetrasubstituted Nitroalkenes and Preliminary Studies of Their Enantioselective Organocatalytic Reduction
Source: Molecules. 2023 Apr 1;28(7):3156. doi: 10.3390/molecules28073156 (PMC10096420; doi:10.3390/molecules28073156)
Supplement: Supplementary file 1 [file molecules-28-03156-s001.zip › molecules-2277489-supplementary.pdf]

Supporting information

Synthesis of Tetrasubstituted Nitroalkenes and Preliminary Studies of Their Enantioselective Organocatalytic Reduction

Table of contents

1. General methods 2
2. Synthesis of tetrasubstituted nitroalkenes **3** 3
  2.1 Initial experiments performed for the synthesis of tetrasubstituted nitroalkenes: 3
  2.2 General procedure for the synthesis of tetrasubstituted nitroalkenes **3** 4
  2.3 Synthesis of acrylates intermediates **2** 4
  2.4 Nitration of acrylates 2 for the synthesis of tetrasubstituted nitroalkenes **3** 6
  2.5 NMR data of tetrasubstituted nitroalkenes **3** 9
3- Synthesis of nitroalkanes **4** 22
  2.1 General procedure for the synthesis of nitroalkanes **4** 22
  2.2 Synthesis of catalyst ***A*** 25
  2.3 Synthesis of Hantzsch ester 25
  2.4 NMR data of tetrasubstituted nitroalkanes **4** 26
  2.5 HPLC data of tetrasubstituted nitroalkanes **4** 34
  2.6 Determination of the absolute configuration of tetrasubstituted nitroalkane ***4a*** 38

1. General methods

All chemical reagents were obtained from commercially available sources such as Sigma Aldrich, TCI chemicals and Combi-Blocks. All solvents were obtained from commercially available sources and had HPLC grade. Reactions were monitored by analytical thin-layer chromatography (TLC) using silica gel 60 F_{254} pre-coated glass plates (0.25 mm thickness) and visualized using UV light. Flash chromatography was carried out on silica gel (230-400 mesh). The enantiomeric excess of the synthetized compounds was measured under the corresponding reported conditions with Agilent 1100 series HPLC. The ^{1}HNMR and ^{13}CNMR experiments were measured using 300MHz Brucker equipment. Proton chemical shifts are showed in ppm (δ) and referenced considering the solvent peak ($CDCl_3$= 7.26 ppm) as internal standard. Carbon chemical shifts are reported in ppm (δ) and referenced using the solvent resonance as the internal standard ($CDCl_3$ = 77.0 ppm). Data are reported as: s = singlet; d = doublet; t= triplet; q= quartet; s= septuplet; m= multiplet. Mass spectra (MS) were carried out at COSPECT (Interdipartimental center of analysis) with mass spectrometer APEX II Xmass software (Brucker Daltonics). Optical rotations were obtained employing a polarimeter at 589 nm using 1 mL cell 1dm long.

2. Synthesis of tetrasubstituted nitroalkenes 3

2.1 Initial experiments performed for the synthesis of tetrasubstituted nitroalkenes:

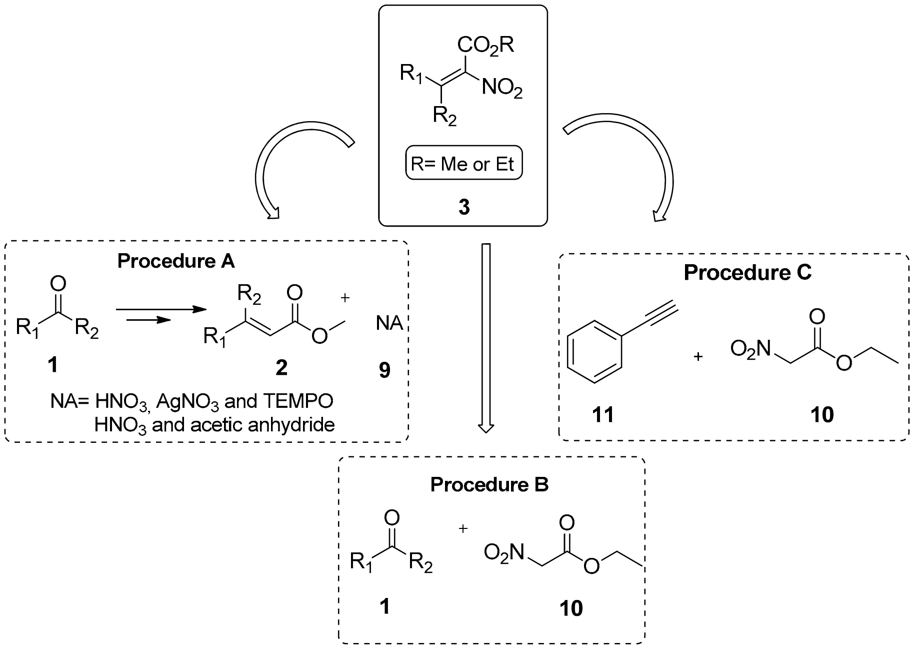

Procedure A

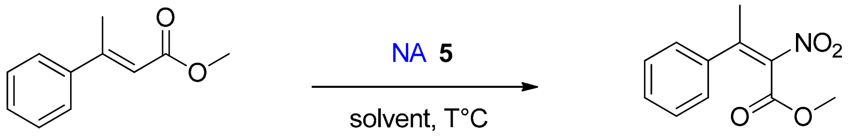

Entry	NA	solvent	T°C	yield
1	HNO_3 fuming	H_2O	0°C→RT	<10%
2	HNO_3 fuming	H_2O	0°C	13%
3	$AgNO_2$, TEMPO	DCE	70°C	No reaction
4	HNO_3, acetic anhydride	TEA, $CHCl_3$	- 10°C→0°C	No reaction

Procedure B

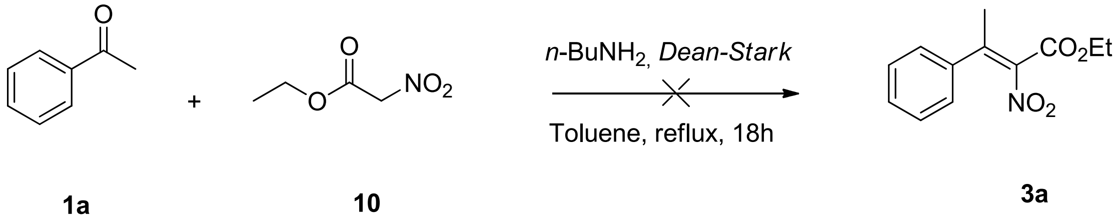

Procedure C

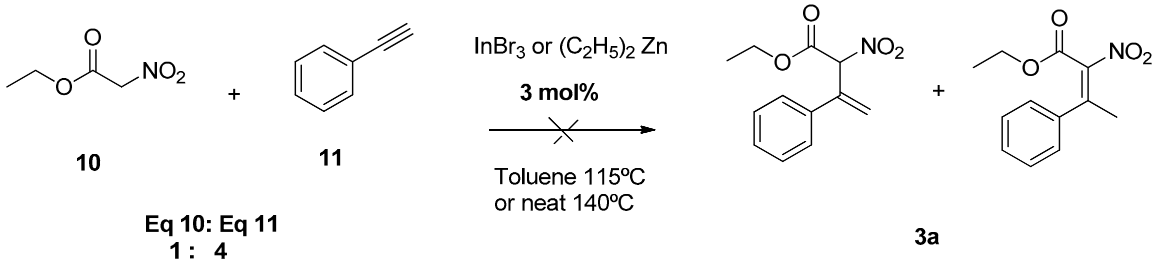

2.2 *General procedure for the synthesis of tetrasubstituted nitroalkenes 3*

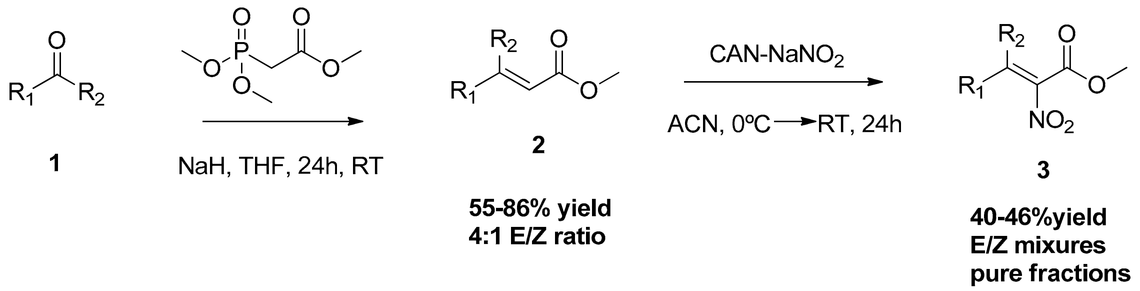

Tetrasubstituted nitroalkenes **3** were synthetized using a two-step procedure: Firstly the formation of an acrylate intermediate **2** by a Horner-Wasdforth-Emmons reaction of an appropriate ketone **1** with trimethylphosphonoacetate and sodium hydride, following by a nitration reaction of this intermediate with a mixture of CAN-$NaNO_2$ as an effective nitration reagent.

2.3 *Synthesis of acrylates intermediates 2*

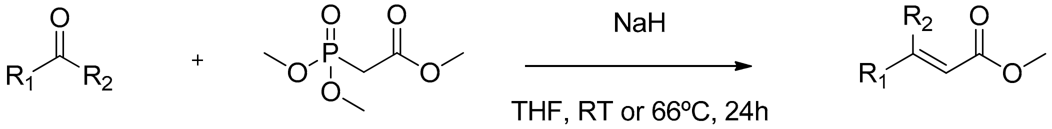

1 **2**

Compounds **2a-f** were synthetized using conditions reported in literature.[9] First, a solution of trimethyl phosphonoacetate (5.21 mmol) in 20 mL of THF was cooled to 0ºC. Then, sodium hydride (5.21 mmol) was added portion wise and the mixture was stirred for 30 min. After this time, the appropriate ketone (4.17 mmol) was added at the same temperature and the reaction mixture was allowed to warm to room temperature and stirred for 24h at the right temperature. Then, 20 mL of saturated solution of ammonium chloride was added dropwise and the mixture was extracted with Et_2O.

The combined organic phases were dried using $MgSO_4$, filtered and concentrated in vacuo. The solvent was eliminated under reduced pressure and the crude was purified using column chromatography and Hexanes/EtOAc as eluent. The ^{1}HNMR of compounds **2a-f** were in agreement with the published ones. Compounds **2a-f** were directly used in the next step after purification.

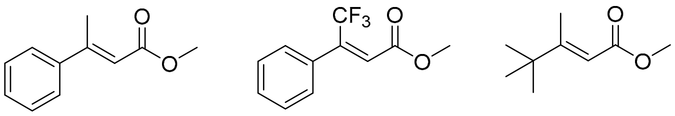

2a
RT
4:1 E/Z ratio
52% yield

2b
RT
3:1 E/Z ratio
79% yield

2c
66ºC
9:1 E/Z ratio
14% yield

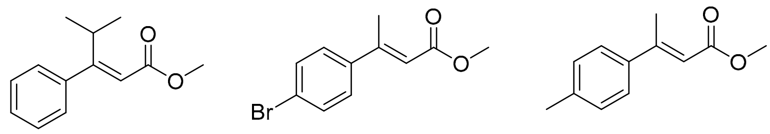

2d
66ºC
2:1 E/Z ratio
33% yield

2e
RT
2:1 E/Z ratio
76% yield

2f
66ºC
3:1 E/Z ratio
86% yield

Figure 1. Structure of the synthetized acrylates 2

2.4 Nitration of acrylates 2 for the synthesis of tetrasubstituted nitroalkenes 3

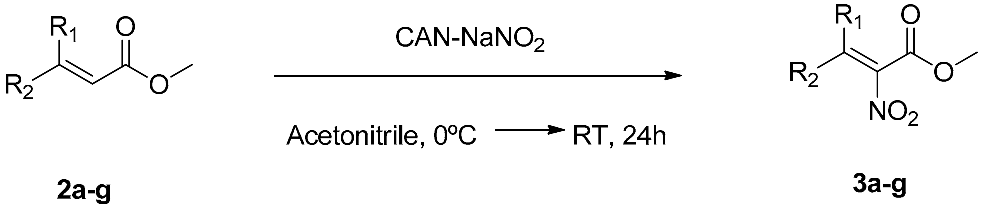

The corresponding acrylates **2a, 2b, 2c, 2d, 2e** and **2f** (5.68 mmol) were dissolved in 50 mL of Acetonitrile and cooled to 0ºC. Then, sodium nitrite (17 mmol) and cerium ammonium nitrate (17 mmol) were added at the same temperature, and the reaction mixture was allowed to warm to room temperature and stirred for 24h. After this time, the reaction was filtered through a pad of celite, and the filtrate was concentrated under reduced pressure. The residue was poured into cold water and extracted with DCM (3 x 50 mL). The combined organic layers were dried using $MgSO_4$, filtered and concentrated in vacuo. The crude was purified by column chromatography using an appropriate mixture of solvents to afford nitroacrylates **2a, 2d, 2f** and **2g** in enriched mixtures of isomers as well as separate fractions of isomers. The reaction of nitration of acrylate **2c** did not lead to the formation of the corresponding nitroacrylate **3c** whereas when the reaction of nitration of acrylate **2b** was performed, the *p*-nitroacrylate was obtained as major compound.

Methyl 2-nitro-3-phenylbut-2-enoate 3a

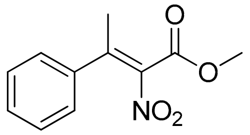

Compound **3a** was obtained in 46% yield as colorless oil after column chromatography using Cyclohexane/Dichloromethane 1:1 as eluent. Both isomers were obtained.

^{1}H NMR first isomer ($CDCl_3$, 300 MHz) 2.55 (s, 3H) 3.85 (s, 3H) 7.21-7.25 (m, 2H) 7.35-7.37 (m, 3H)

^{1}H NMR second isomer ($CDCl_3$, 300 MHz) 2.35 (s, 3H) 3.66 (s, 3H) 7.26-7.29 (m, 2H) 7.42-7.45 (m, 3H)

^{13}C NMR ($CDCl_3$, 300MHz) 22.82, 52.80, 126.60, 128.53, 129.47, 137.20, 141.84, 149.26, 159.96

HR-MS (M^+ + 23) predicted values m/z = 244. 0688 (100%); 245.0722 (11%) experimental values m/z = 244.0591 (100%) ; 245.0622 (11%)

Methyl 4-methyl-2-nitro-3-phenylpent-2-enoate 3d

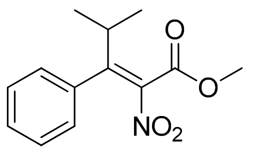

Compound **3d** was obtained in 25% yield as colorless oil after column chromatography using Cyclohexane/Diethyl ether (from 99:1 to 95:5) as eluent. In this case, one isomer pure and a mixture of both isomers were isolated

^{1}H NMR ($CDCl_3$ 300 MHz) 1.04 (d, 6H) 3.87 (s, 3H) 3.93-4.02 (s, 1H) 7.07-7.10 (m, 2H) 7.34-7.36 (3H)

^{13}C NMR ($CDCl_3$, 300MHz) 20.37, 29.57, 52.97, 127.38, 127.88, 132.37, 141.55, 158.48, 159.51

HR-MS (M^+ + 23) predicted values m/z = 272.1001 (100%) ; 273.1035 (14.1) experimental values m/z = 272.0893 (100%); 273.0926 (14.1) (M^+ + Na)

Methyl 3-(4-bromophenyl)-2-nitrobut-2-enoate 3e

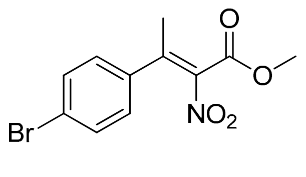

Compound **3e** was obtained as colorless oil in 47% yield after column chromatography in Cyclohexane / Dichloromethane 1:1. Both isomers were obtained.

^{1}H NMR first isomer ($CDCl_3$, 300 MHz) 2.54 (s, 3H) 3.87 (s, 3H) 7.11 (d, 2H) 7.52 (d, 2H)

^{1}H NMR second isomer (CDCl$_3$, 300 MHz) 2.28 (s, 3H) 3.66 (s, 3H) 7.13 (d, 2H) 7.55 (d, 2H)

^{13}C NMR (CDCl$_3$, 300 MHz) 22.90, 53.14, 124.00, 128.45, 131.97, 136.10, 142.06, 148.07, 159.67

HR-MS (M$^+$ + 23) predicted values m/z = 321.9793 (100%) ; 323.9773 (97.3%) ; 322.9827 (11.9%); 324.9806 (11.6%) experimental values m/z = 321.9691 (100%) ; 323.9670 (97.3%) ; 322.9723 (11.9%) ; 324.9703 (11.6%)

Methyl 2-nitro-3-(p-tolyl)-2-butenoate 3f

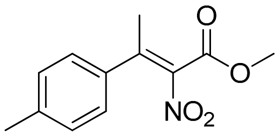

Compound **3f** was obtained as colorless oil in 48% yield after purification with column chromatography and Cyclohexane/ Dichloromethane 1:1. One isomer and a mixture of both isomers were obtained.

^{1}H NMR (CDCl$_3$, 300 MHz) 2.35 (s, 3H) 2.55 (s, 3H) 3.86 (s, 3H) 7.12-7.20 (m, 4H)

^{13}C NMR (CDCl$_3$, 300 MHz) 21.27, 21.58, 52.89, 125.95, 129.55, 134.78, 139.95, 150.44, 160.00

2.5 NMR data of tetrasubstituted nitroalkenes 3

2.5.1 Compound 3a

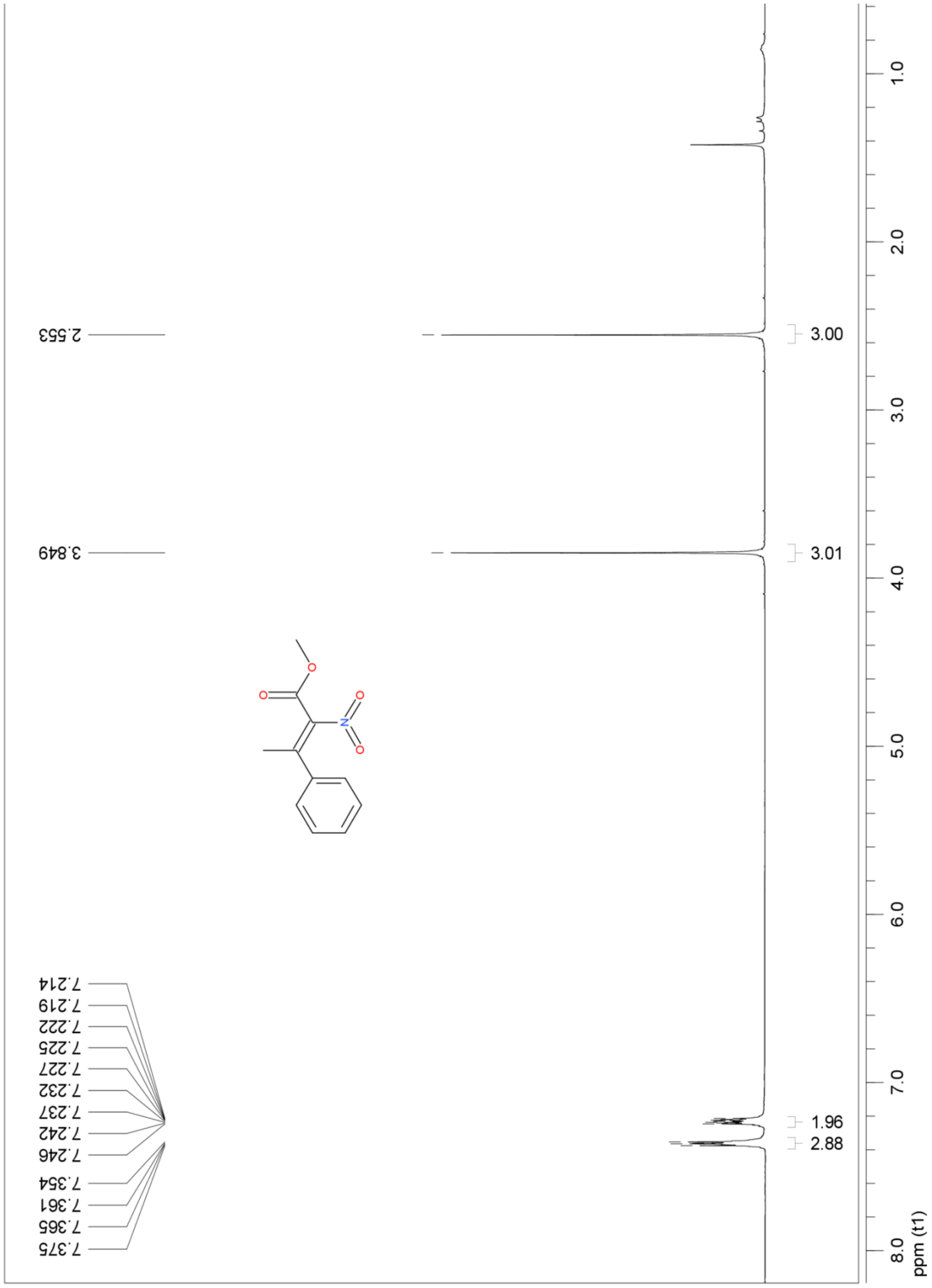

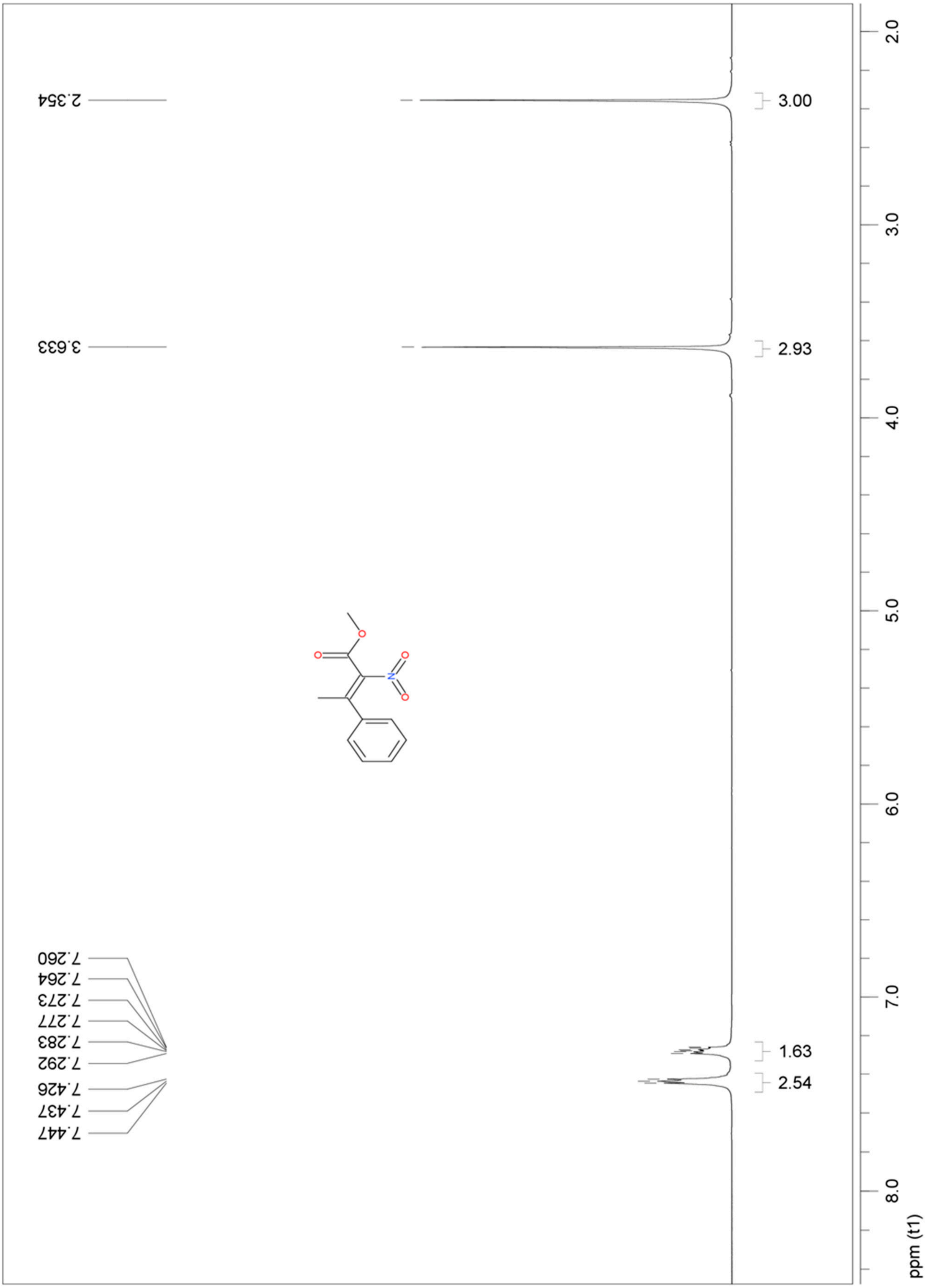

2.354
3.633
7.260
7.264
7.273
7.277
7.283
7.292
7.426
7.437
7.447
3.00
2.93
1.63
2.54
2.0
3.0
4.0
5.0
6.0
7.0
8.0
ppm (t1)

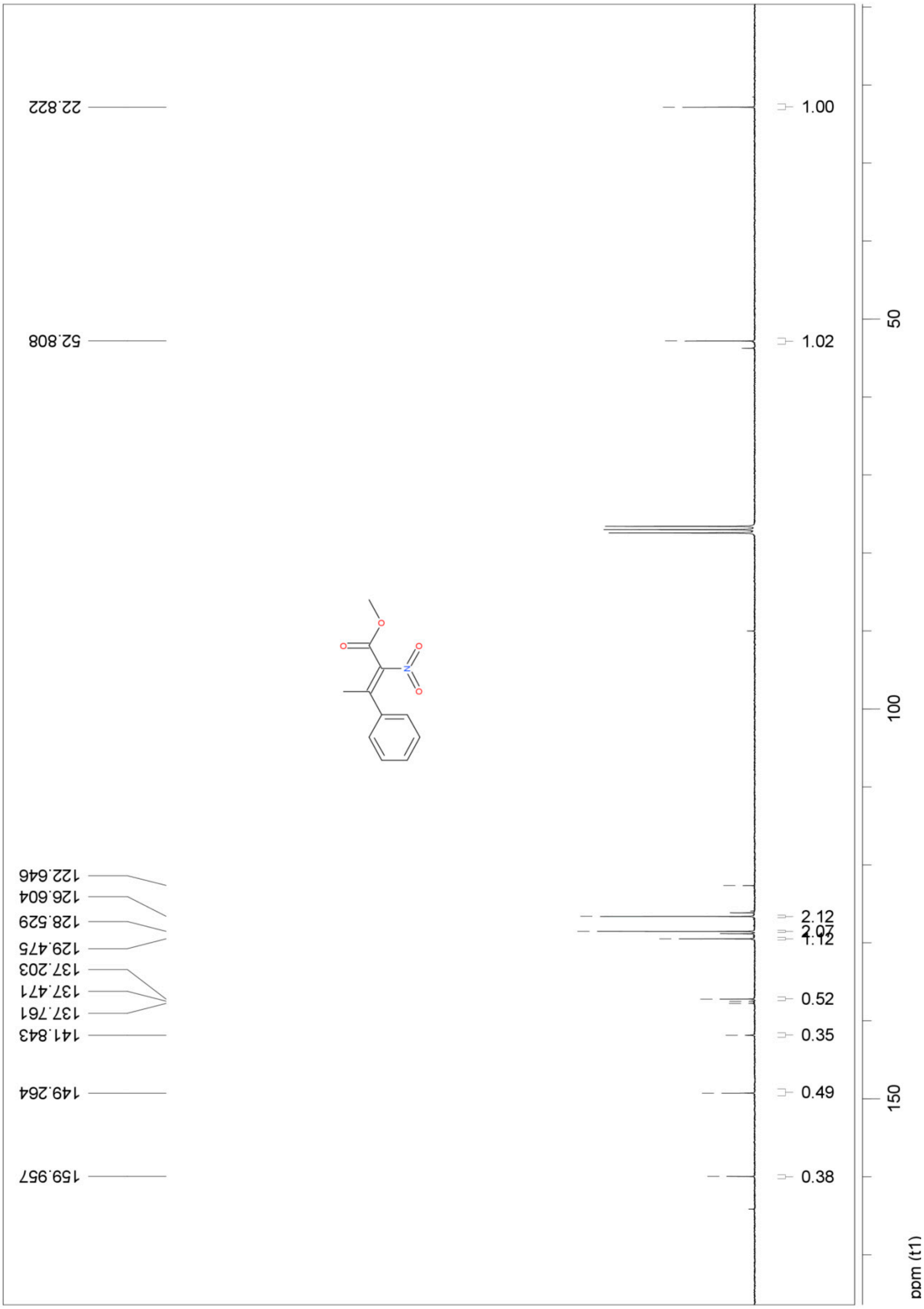
22.822
52.808
122.646
126.604
128.529
129.475
137.203
137.471
137.761
141.843
149.264
159.957
1.00
1.02
2.12
2.07
1.12
0.52
0.35
0.49
0.38
50
100
150
ppm (t1)

HR-MS Compound 3a

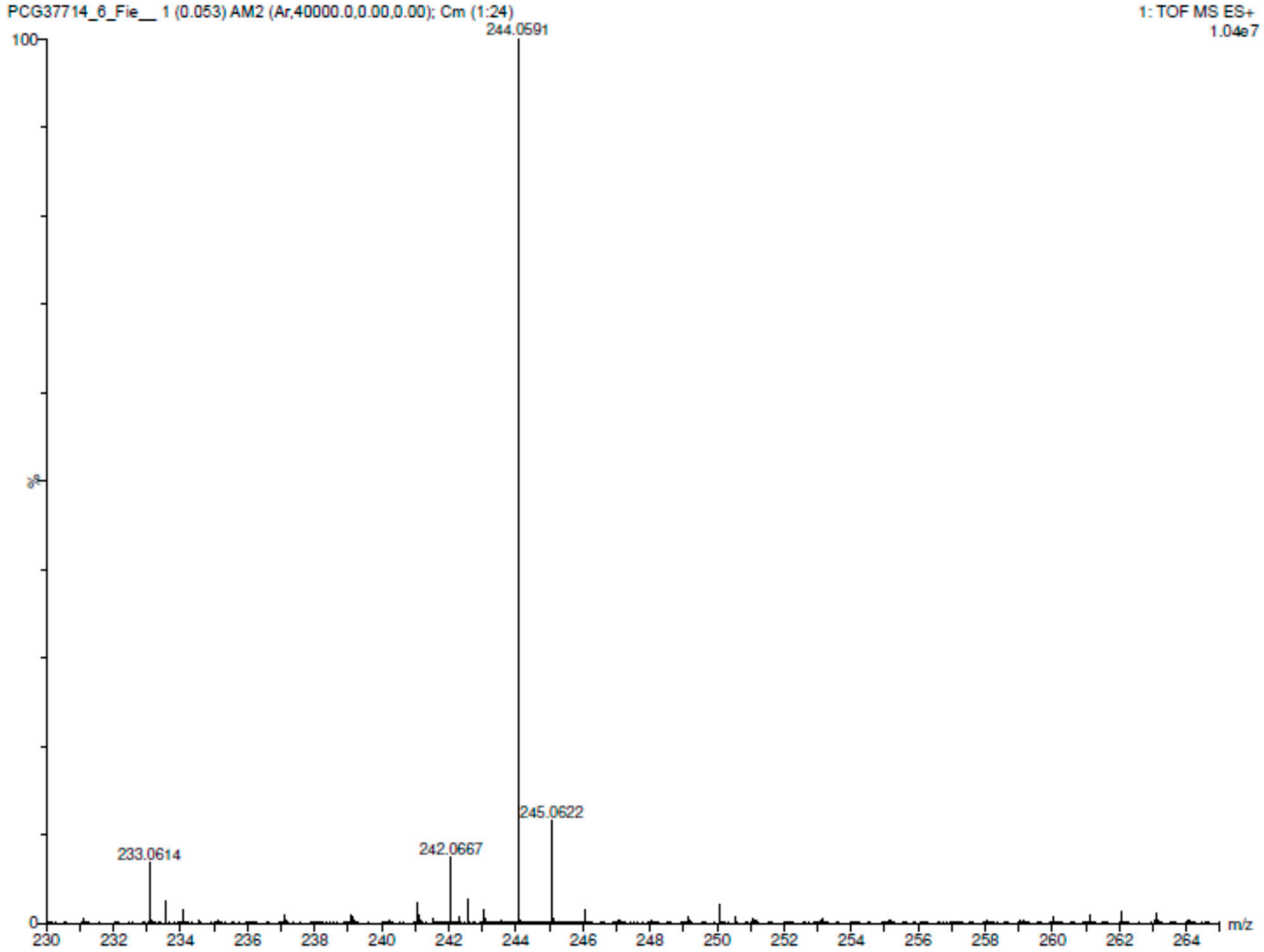

Compound 3d

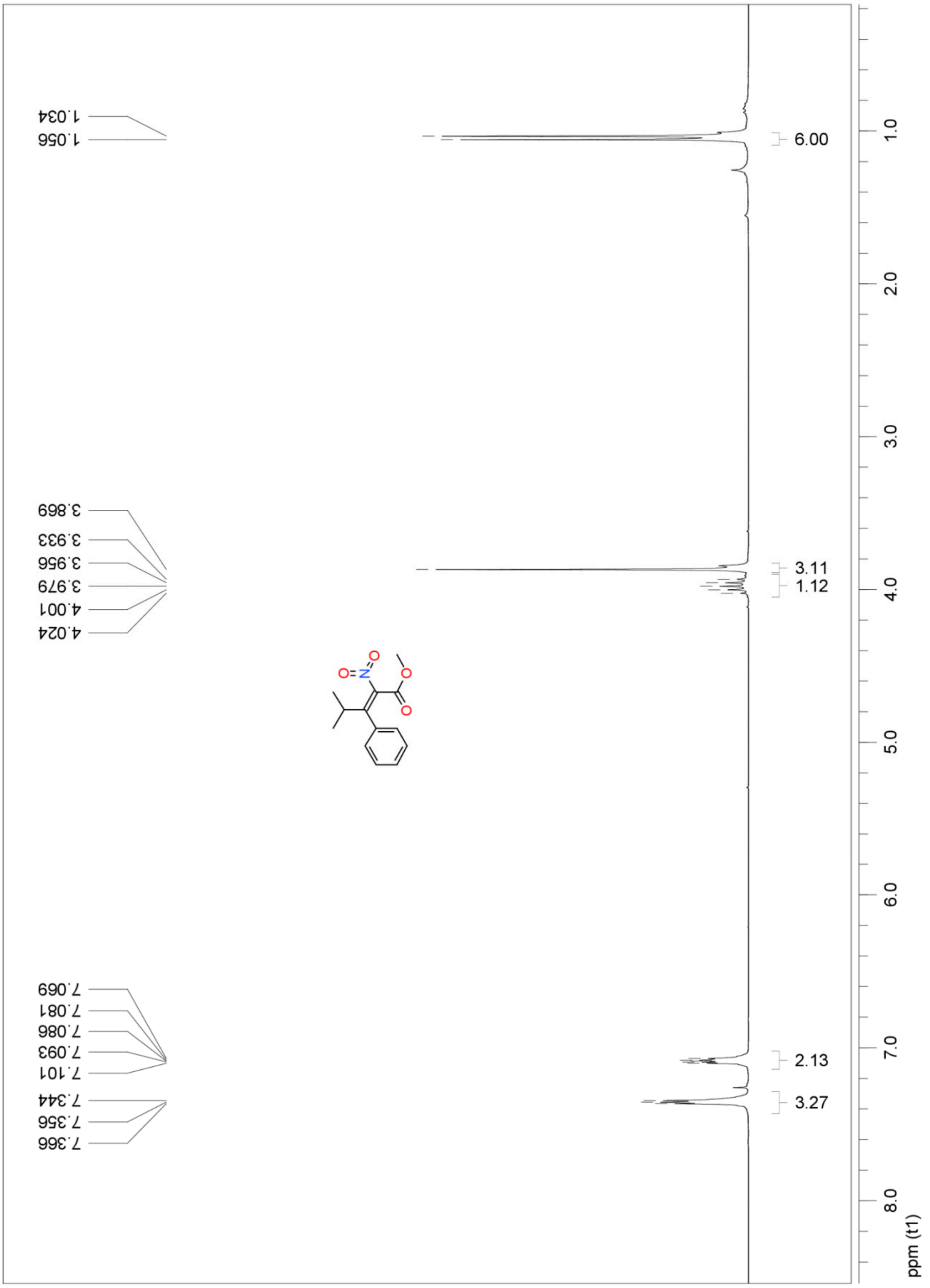
1.034
1.056
3.869
3.933
3.956
3.979
4.001
4.024
7.069
7.081
7.086
7.093
7.101
7.344
7.356
7.366
6.00
3.11
1.12
2.13
3.27
1.0
2.0
3.0
4.0
5.0
6.0
7.0
8.0
ppm (t1)

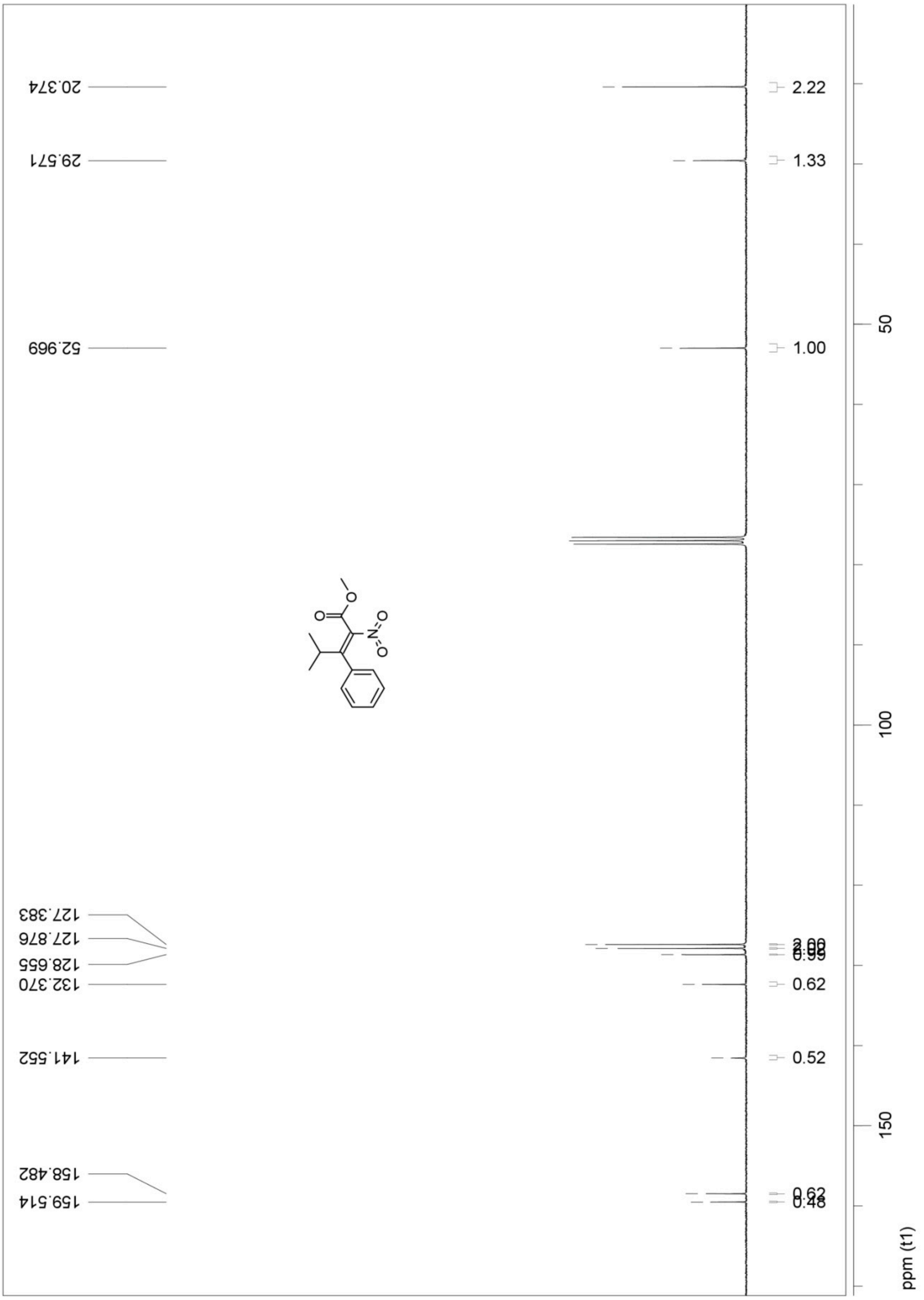
20.374
29.571
52.969
127.383
127.876
128.655
132.370
141.552
158.482
159.514
2.22
1.33
1.00
0.62
0.52
50
100
150
ppm (t1)

HR-MS Compound 3d

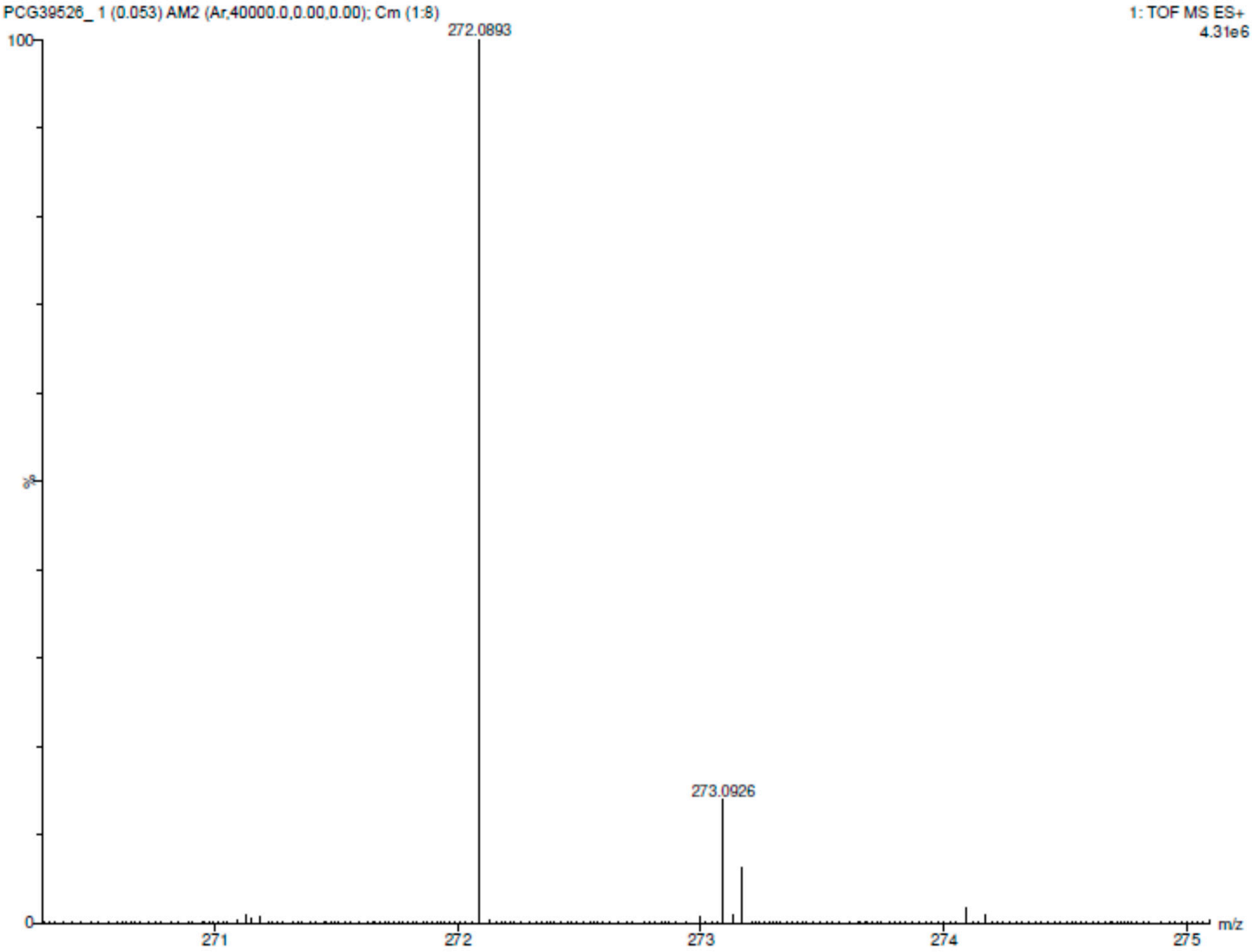

Compound 3e

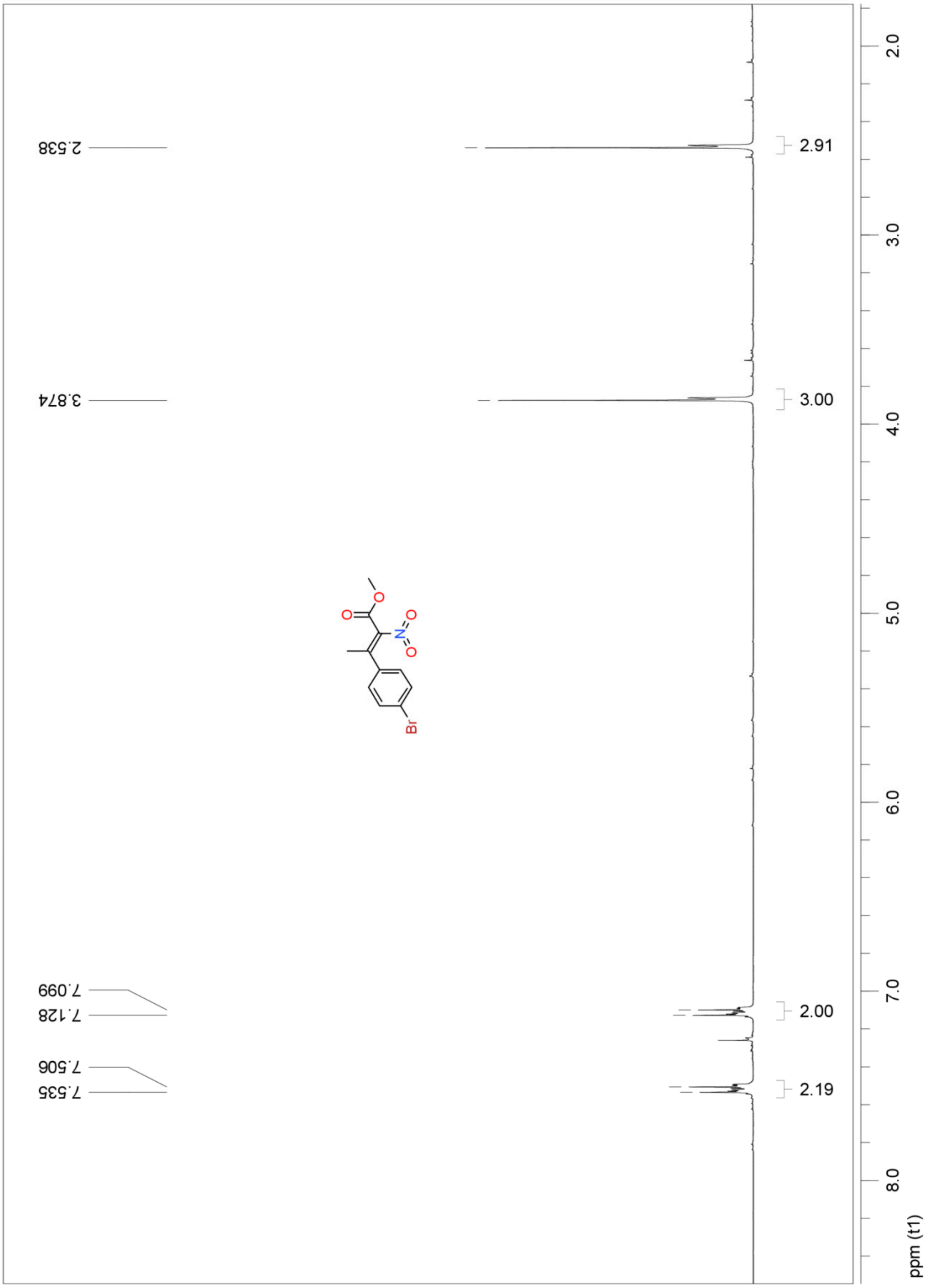

2.538
3.874
7.099
7.128
7.506
7.535
2.91
3.00
2.00
2.19
2.0
3.0
4.0
5.0
6.0
7.0
8.0
ppm (t1)
Br

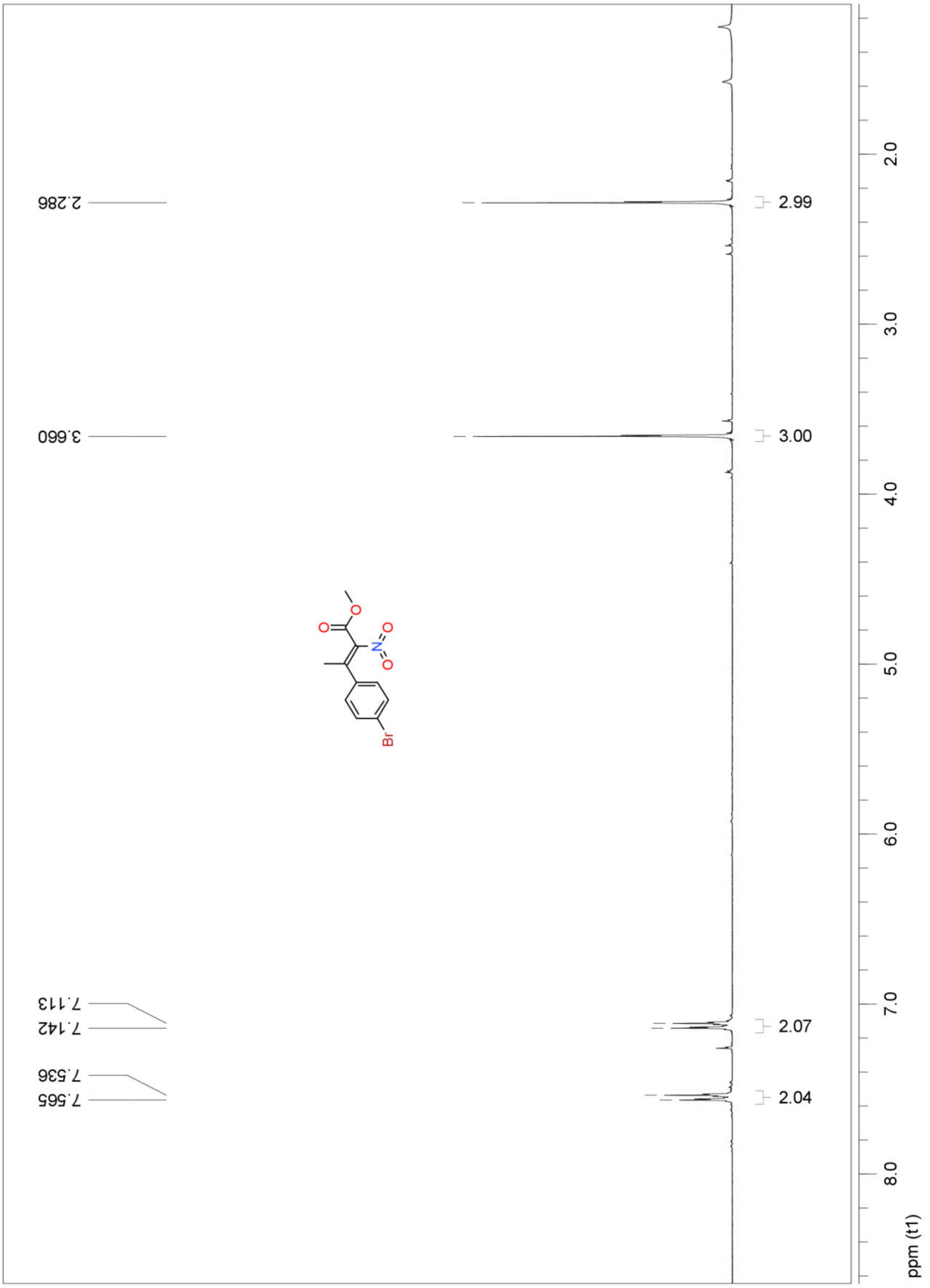
2.286
3.660
7.113
7.142
7.536
7.565
2.99
3.00
2.07
2.04
2.0
3.0
4.0
5.0
6.0
7.0
8.0
ppm (t1)
O
O
N
O
O
Br

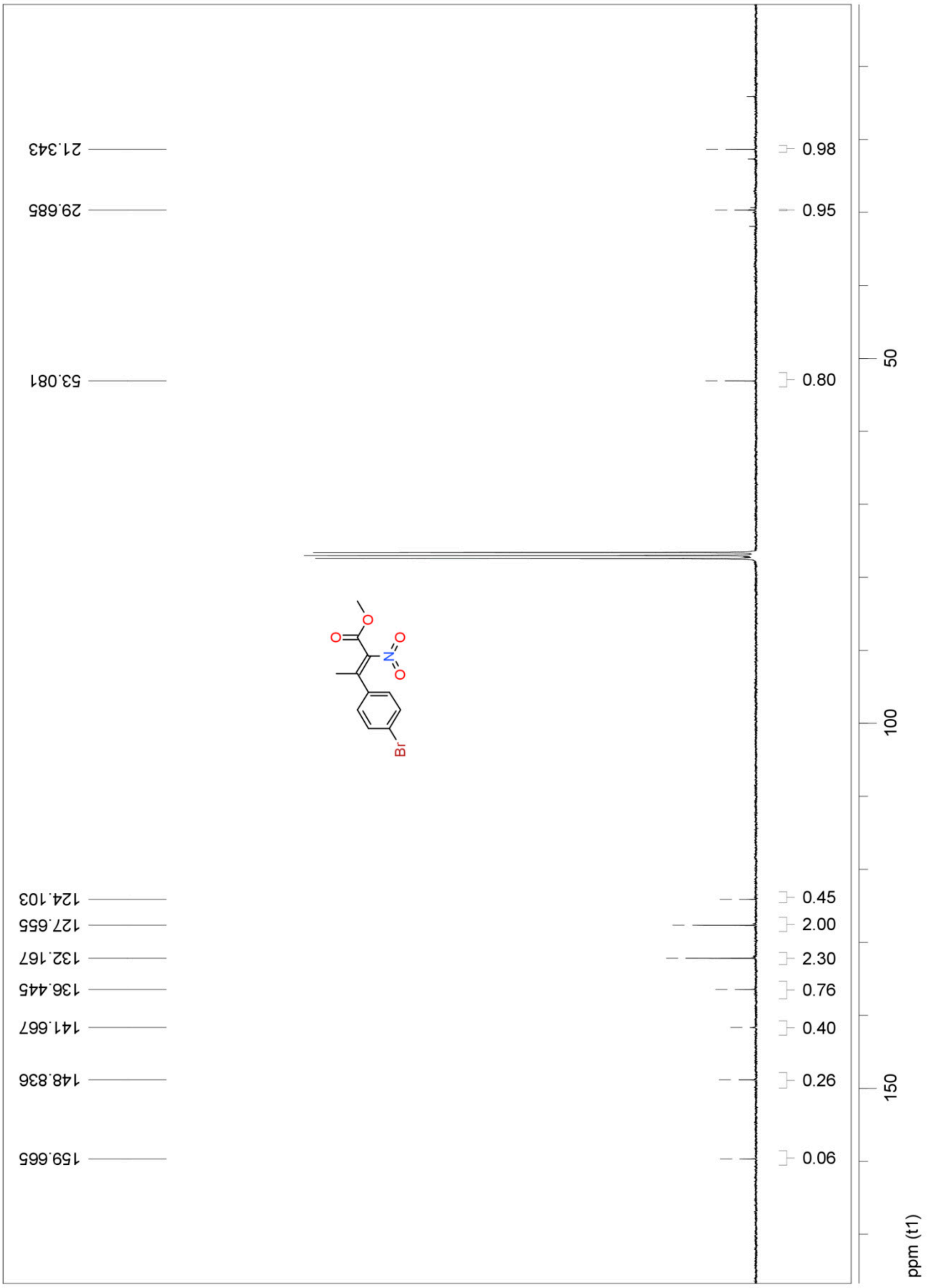
21.343
29.685
53.081
124.103
127.655
132.167
136.445
141.667
148.836
159.665
0.98
0.95
0.80
0.45
2.00
2.30
0.76
0.40
0.26
0.06
50
100
150
ppm (t1)
Br

HR-MS Compound 3e

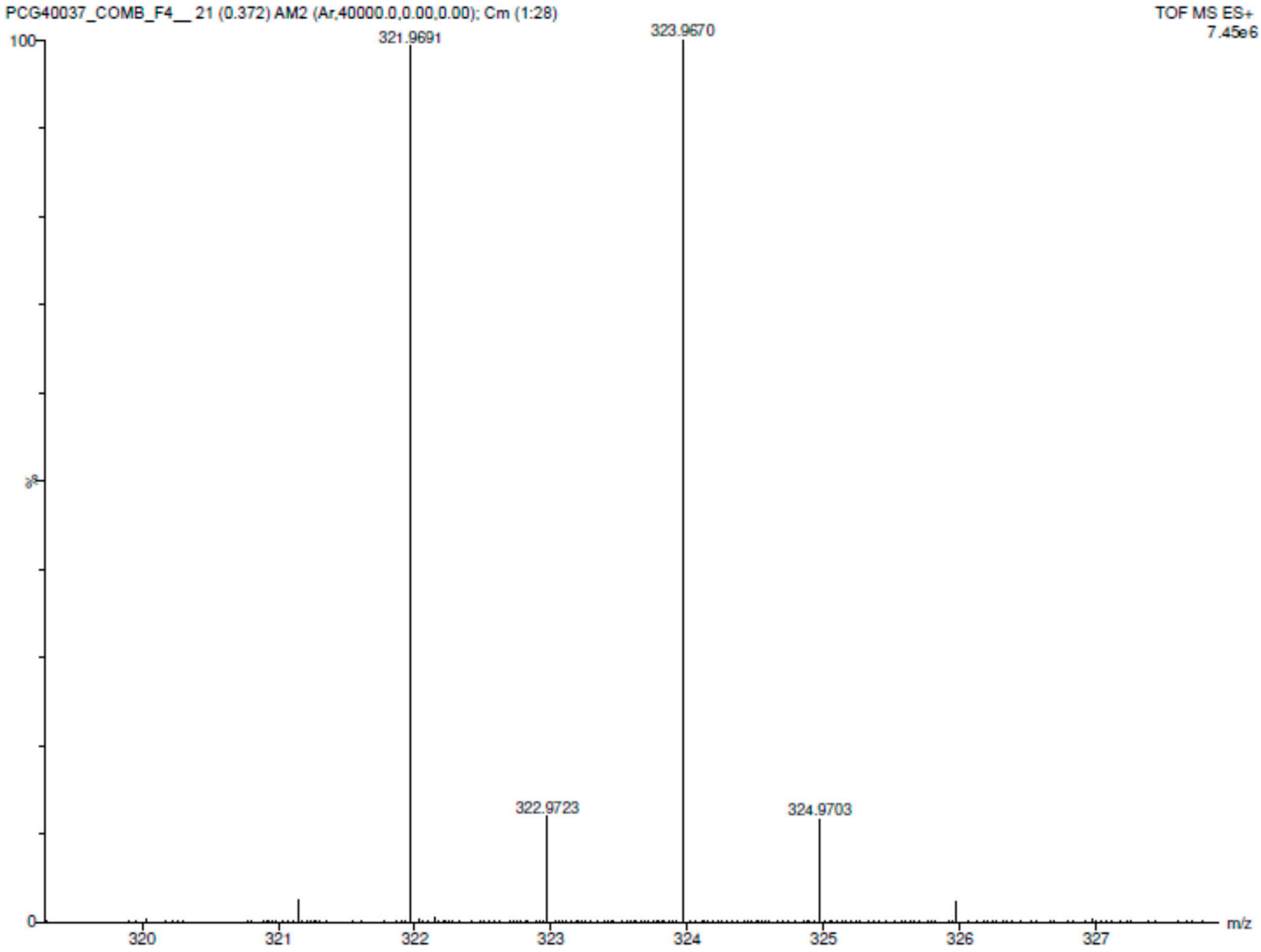

Compound 3f

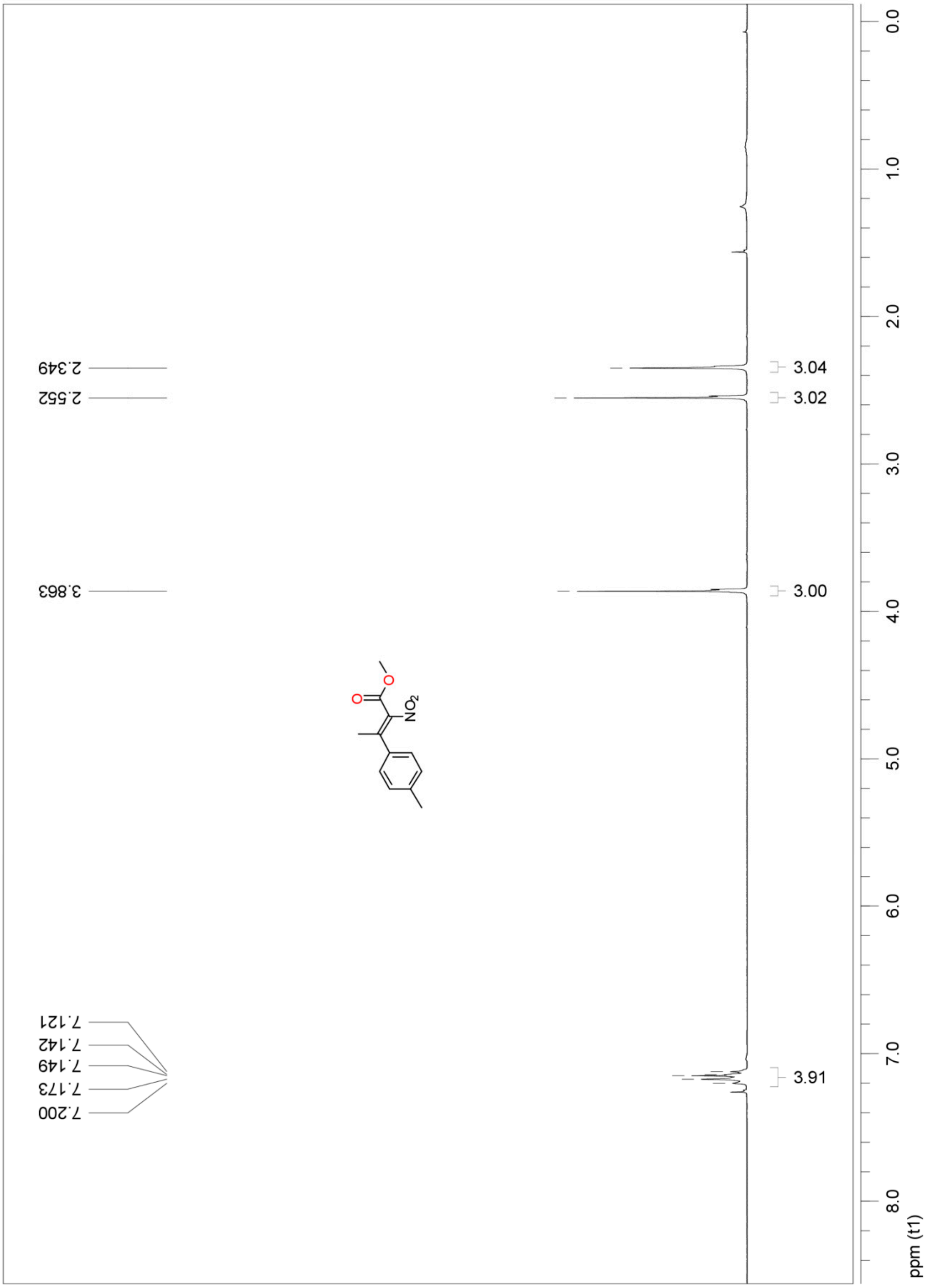
2.349
2.552
3.863
7.121
7.142
7.149
7.173
7.200
3.04
3.02
3.00
3.91
0.0
1.0
2.0
3.0
4.0
5.0
6.0
7.0
8.0
ppm (t1)
NO2

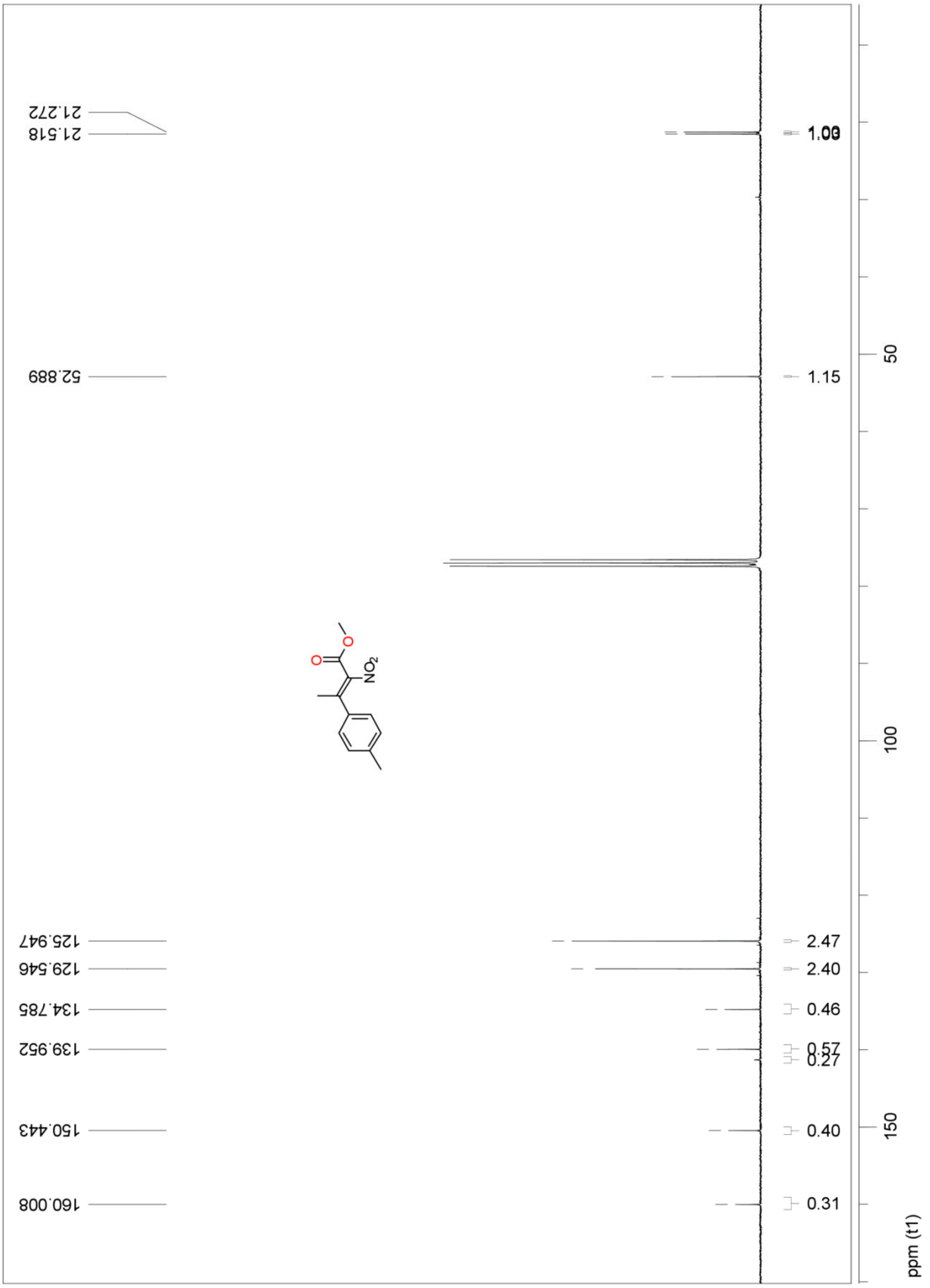
21.272
21.518
52.889
125.947
129.546
134.785
139.952
150.443
160.008
NO2
1.00
1.15
2.47
2.40
0.46
0.57
0.27
0.40
0.31
50
100
150
ppm (t1)

3- Synthesis of nitroalkanes 4

3.1 General procedure for the synthesis of nitroalkanes 4 by reduction of compounds 3.

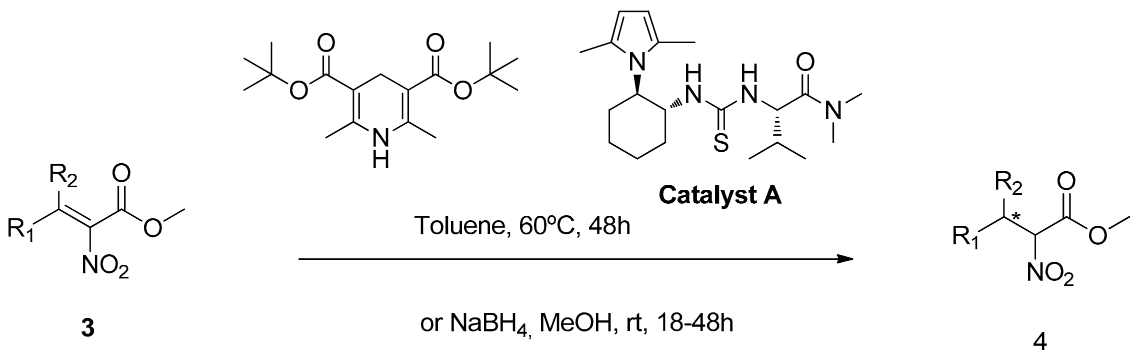

Synthesis of racemic compounds 4: A solution of the corresponding nitroalkene **3** (1 eq, 0,5 mmol) in MeOH (0.5 mL, 0.2M) was cooled to 0ºC. Then, $NaBH_4$ (2 eq, 1 mmol) was added and the mixture was allowed to warm to room temperature and stirred at RT for 18-48h. After this time, a saturated solution of ammonium chloride was added and the mixture was extracted with dichloromethane. The combined organic layers were dried using $MgSO_4$, filtered and concentrated in vacuo. The crude was purified using column chromatography or preparative HPLC purification.

Synthesis of enantioenriched compounds 4: To a stirred solution of nitroalkenes **3** in Toluene (0,3 mmol 0.3M), catalyst **A** (10 mol%) and Hanztsch ester (1.2 eq, 0,36 mmol) were added. The reaction mixture was heated at 60ºC for 24h. Then, the mixture was allowed to warm to room temperature and the solvent was eliminated under reduced pressure, and the crude was purified using column chromatography and an appropriate mixture of eluents.

Methyl 2-nitro-3-phenylbutanoate 4a

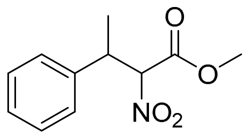

Yield 85%

Compound **4a** was obtained as colorless oil and a 1:1 mixture of *syn/anti* products after column chromatography using Cyclohexane/Dichloromethane

7:3 as eluent. Enantiomeric excess was measured using chiral HPLC column Phenomenex-Cellulose 5_Hexane_IPA_98_2_0.5 mL/min

^{1}H NMR ($CDCl_3$, 300 MHz) 1.35-1.41 (m, 6H) 3.54 (s, 3H) 3.76-3.78 (m, 2H) 3.84 (s, 3H) 5.22-5.32 (m, 2H) 7.20-7.31 (m, 10 H)

^{13}C NMR ($CDCl_3$, 300 MHz) 18.30, 29.65, 41.31, 53.40, 93.49, 127.04, 127.85, 128.40, 129.00, 139.96, 164.24.

HR-MS predicted values m/z = 223.0845 (100%); experimental values m/z = 246.0742 (100%) (M^+ + Na).

Methyl 4-methyl-2-nitro-3-phenylpentanoate 4d

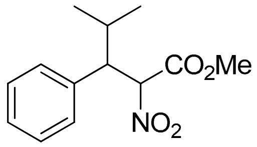

Yield 32%

Compound **4d** was obtained as colorless oil and a 1:1 mixture of *syn/anti* products after column chromatography using Cyclohexane/ Diethyl ether 9:1 as eluent. Enantiomeric excess was measured using chiral HPLC column Phenomenex-Cellulose 5_Hexane_IPA_95_5_1 mL/min

^{1}H NMR ($CDCl_3$, 300 MHz) 0.83-0.88 (m, 12H) 3.50 (s, 3H) 3.60-3.65 (m, 2H) 3.86 (s, 3H) 5.59-5.67 (m, 2H) 7.14-7.18 (m, 4H) 7.26-7.31 (m, 6H)

^{13}C NMR ($CDCl_3$, 300 MHz) 17.82, 20.95, 29.19, 52.19, 53.18, 90.58, 127.71, 128.25, 129.15, 134.90, 163.74, 164.53

HR-MS predicted values m/z = 251.1158 (100%); experimental values m/z = 274.1050 (100%) (M^+ + Na).

Methyl 3-(4-bromophenyl)-2-nitrobutanoate 4e

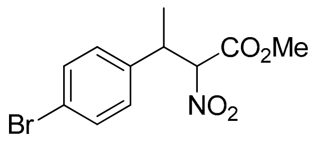

Yield 59%

Compound **4e** was obtained as colorless oil and a 1:1 mixture of *syn*/*anti* products after column chromatography using Cyclohexane /DCM 7:3 as eluent. Enantiomeric excess was measured using chiral HPLC column Phenomenex-Cellulose 5_Hexane_IPA_95:5_1 mL/min

^{1}H NMR ($CDCl_3$, 300 MHz) 1.35-1.41 (m, 6H) 3.61 (s, 3H) 3.73 (m, 2H) 3.86 (s, 3H) 5.20-5.26 (m, 2H) 7.11-7.14 (m, 4H) 7.43-7.46 (m, 4H)

^{13}C NMR ($CDCl_3$, 300 MHz) 18.01, 40.60, 53.38, 92.73, 121.81, 129.02, 129.40, 132.06, 137.98, 138.77, 163.79

HR-MS predicted values m/z = 300.9950 (100%); experimental values m/z = 323.9853 (100%) (M^+ + Na).

Methyl 2-nitro-3-(p-tolyl)-butanoate 4f

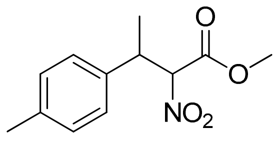

Yield chiral 51%

Compound **4f** was obtained as colorless oil and a 1:1 mixture of *syn*/*anti* products after column chromatography using Cyclohexane/DCM as eluent. Enantiomeric excess was measured using chiral HPLC column Phenomenex-Cellulose 5_Hexane_IPA_95:5_1 mL/min.

^{1}H NMR ($CDCl_3$, 300 MHz) 1.35-1.41 (m, 6H), 2.31 (d, 6H), 3.59 (s, 3H) 3.74-3.80 (m, 2H) 3.86 (, 3H) 5.22-5.29 (m, 2H) 7.12 (s, 8H)

^{13}C NMR ($CDCl_3$, 300 MHz) 18.11, 20.96, 40.87, 53.08, 93.29, 127.15, 127.55, 129.61, 136.09, 136.95, 137.54, 137.58, 164.15

HR-MS predicted values m/z = 237.1001 (100%); experimental values m/z = 260.1005 (100%) (M^+ + Na).

2.1 Synthesis of catalyst A

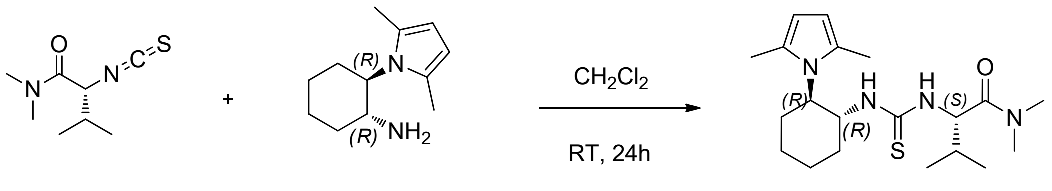

12 **13** **80% yield** **Catalyst A**

Catalyst A was synthesized according with literature procedure.[1] Working under inert atmosphere, a 0.1M solution of compound **12** (1.2 eq., 0.18 mmol) in dichloromethane was added to a solution of compound **13** (1 eq., 0.15 mmol) in dichlormethane at room temperature. The reaction mixture was stirred 48 hours and subsequently concentrated under reduced pressure. The product was purified though flash column chromatography on silica gel. ^{1}HNMR of Catalyst A, was in agreement with the published one.

2.2 Synthesis of Hantzsch ester

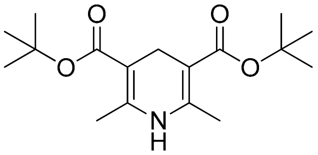

The *tert*-butyl Hantzsch ester was synthetized according to a procedure found in the literature.[2] A solution of paraformaldehyde (0.75 g, 25 mmol), *tert*-butyl acetoacetate (8.25 mL, 50 mmol), and aqueous NH_4OH (15 mL of a 5 M solution, 75 mmol) in Ethanol (20 mL) was heated at reflux (oil bath at 85°C) for 2 h. The mixture was then cooled to RT, poured into ice-water (75 mL) and extracted with Et_2O (100 mL). The ether phase was washed successively with 10% aqueous solution of NaOH (50 mL), water (50 mL), 5% aqueous solution of HCl (50 mL) and water (50 mL). The ether solution was dried over $MgSO_4$ and filtered. The solvent was removed *in vacuum* to afford a yellow solid. The crude product was crystallized with MeOH (about 6-8 mL). To avoid oxidation of the dihydropyridine to the corresponding pyridine derivative, the solubilization of the crude product with MeOH was promptly done and the

---

[1] Massolo, E., Benaglia, M., Orlandi, M., Rossi, S Celentano, G. *Enantioselective Organocatalytic Reduction of β-Trifluoromethyl Nitroalkenes: An Efficient Strategy for the Synthesis of Chiral β-Trifluoromethyl Amines. Chem Eur J* **2015**, *21*, 3589-3595

[2] Ferraro, A., Bernardi, L. and Fochi, M. *Organocatalytic Enantioselective Transfer Hydrogenation of β-Amino Nitroolefins Adv Synth Catal*, 2016, 358, 1561-1565

recrystallization was carry out under nitrogen atmosphere for up to 2 h. The *tert*-butyl Hantzsch ester was obtained as yellow solid in 45% yield.

2.3 NMR data of nitroalkanes 4

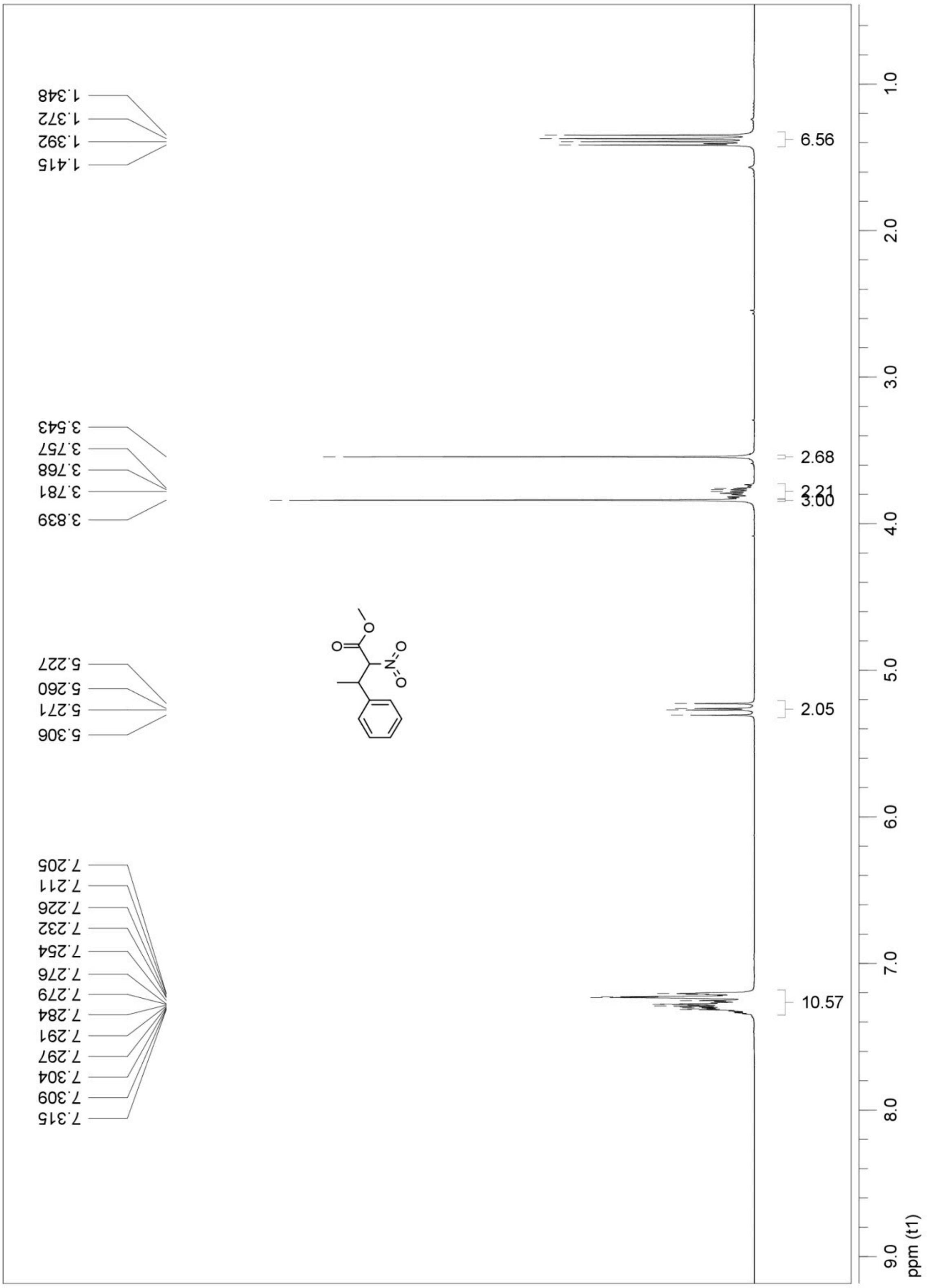

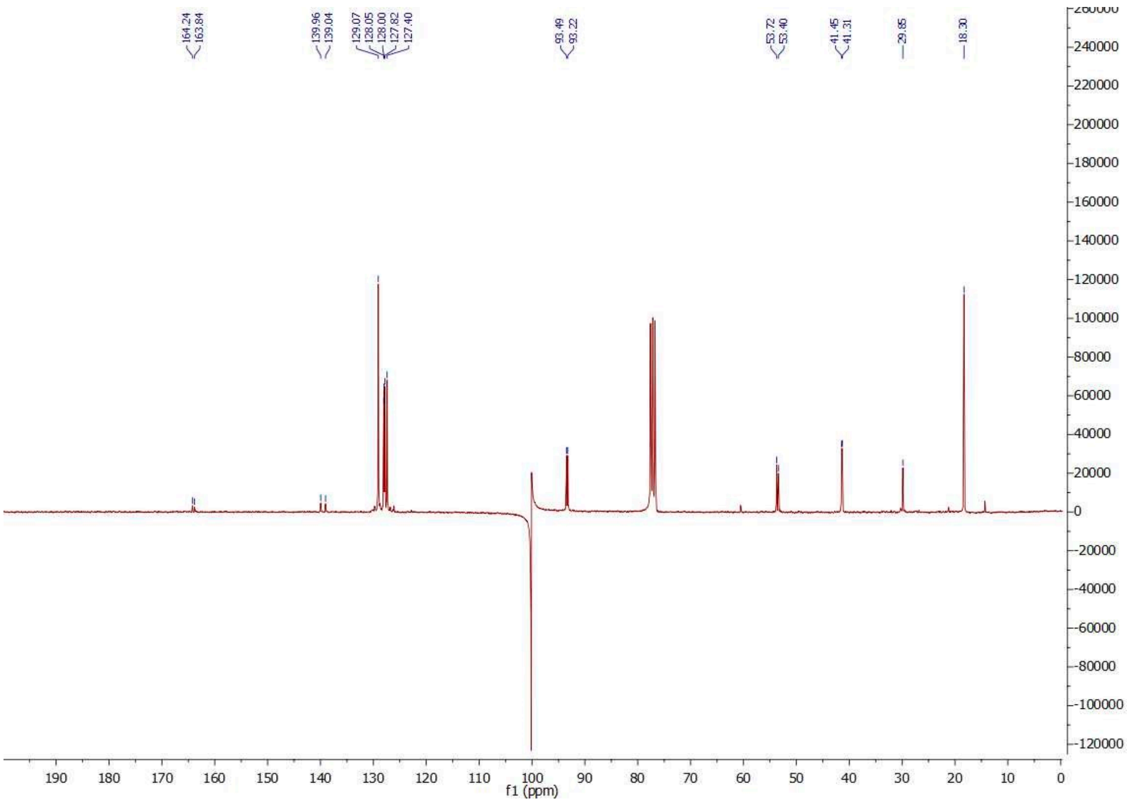

164.24
163.84
139.96
139.04
129.07
128.05
128.00
127.82
127.40
93.49
93.22
53.72
53.40
41.45
41.31
29.85
18.30
240000
220000
200000
180000
160000
140000
120000
100000
80000
60000
40000
20000
0
-20000
-40000
-60000
-80000
-100000
-120000
190 180 170 160 150 140 130 120 110 100 90 80 70 60 50 40 30 20 10 0
f1 (ppm)

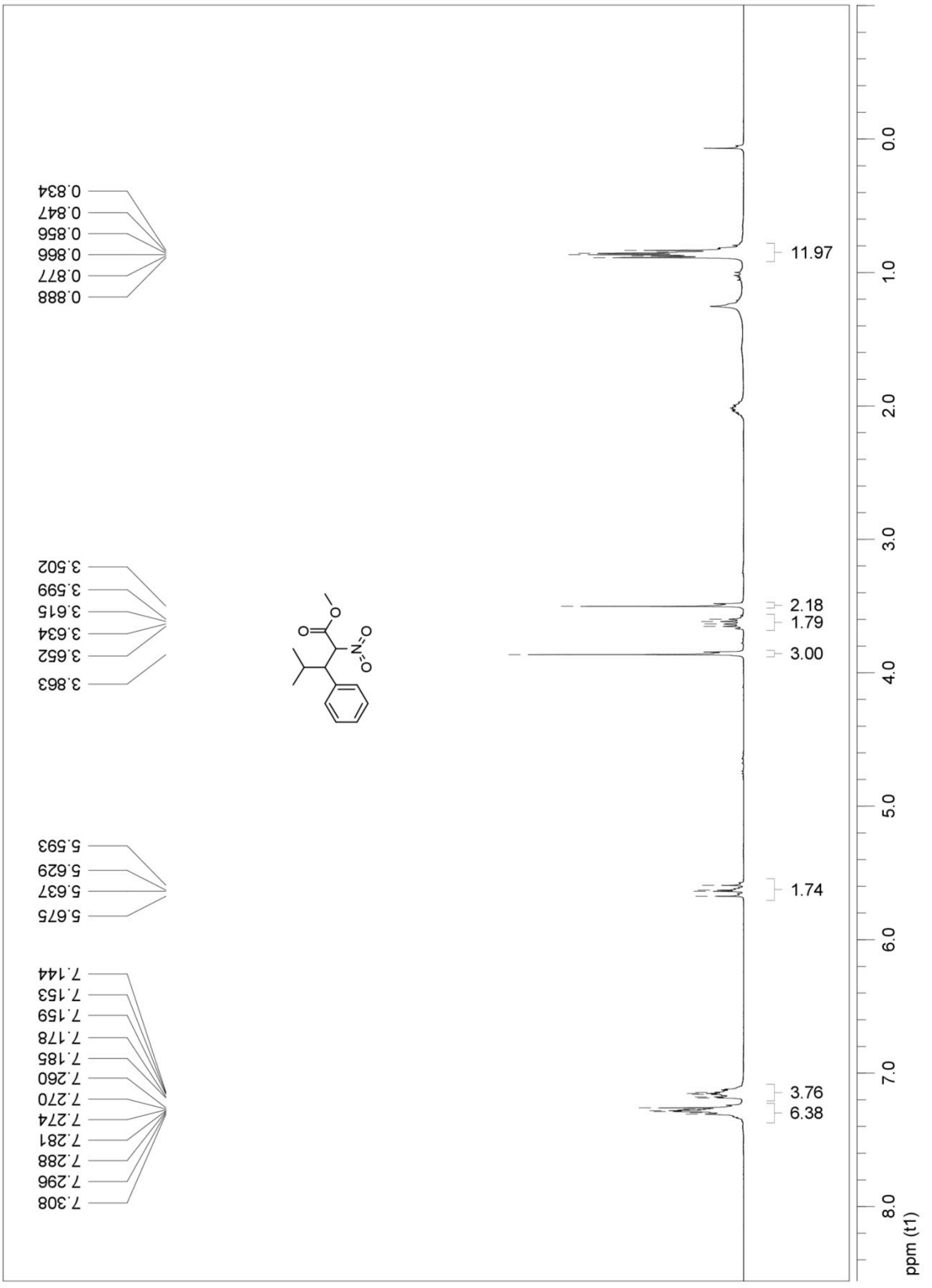

0.834
0.847
0.856
0.866
0.877
0.888
3.502
3.599
3.615
3.634
3.652
3.863
5.593
5.629
5.637
5.675
7.144
7.153
7.159
7.178
7.185
7.260
7.270
7.274
7.281
7.288
7.296
7.308
11.97
2.18
1.79
3.00
1.74
3.76
6.38
ppm (t1)

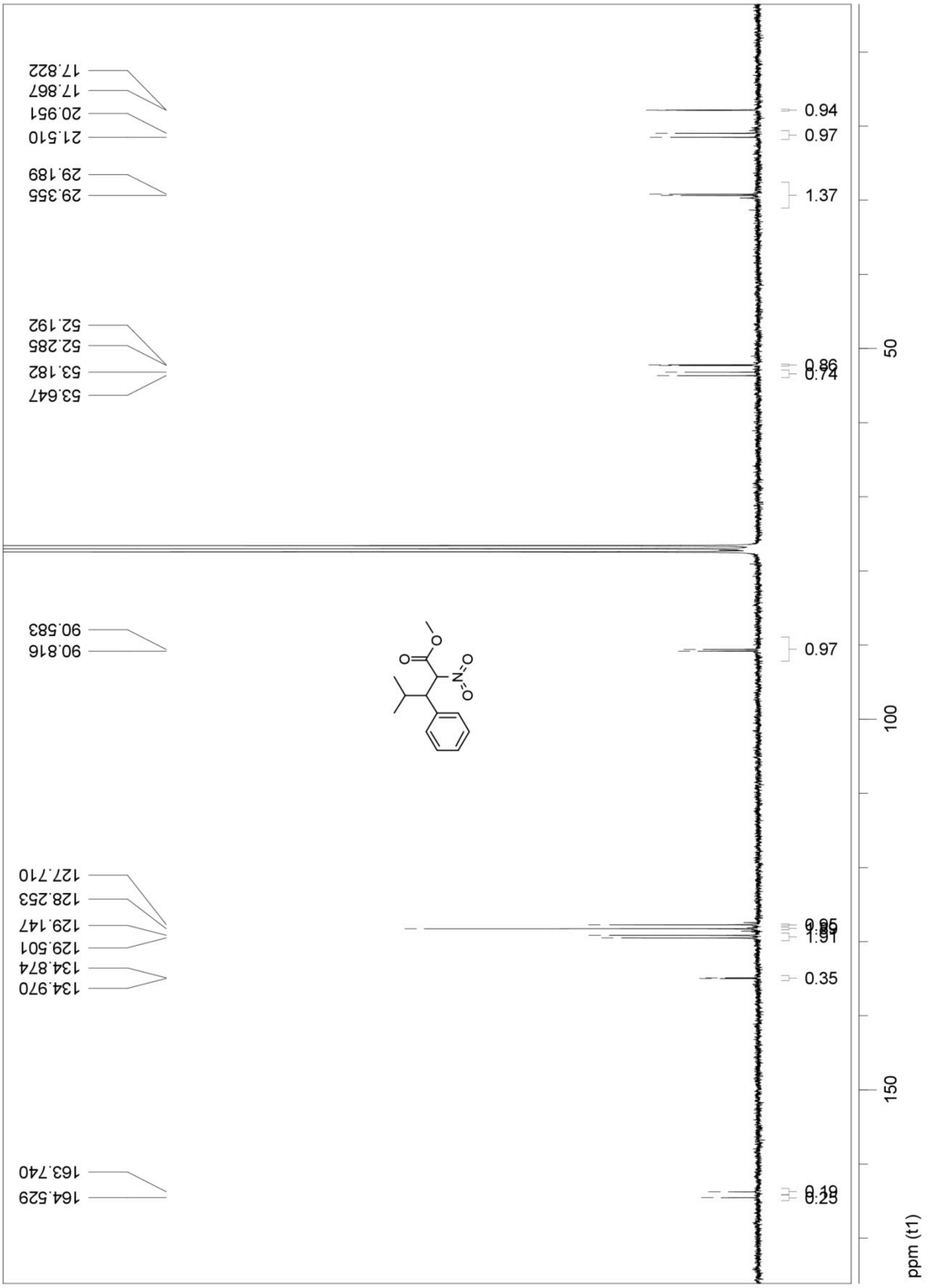
17.822
17.867
20.951
21.510
29.189
29.355
52.192
52.285
53.182
53.647
90.583
90.816
127.710
128.253
129.147
129.501
134.874
134.970
163.740
164.529
0.94
0.97
1.37
0.86
0.74
0.97
1.91
0.35
0.19
0.25
50
100
150
ppm (t1)

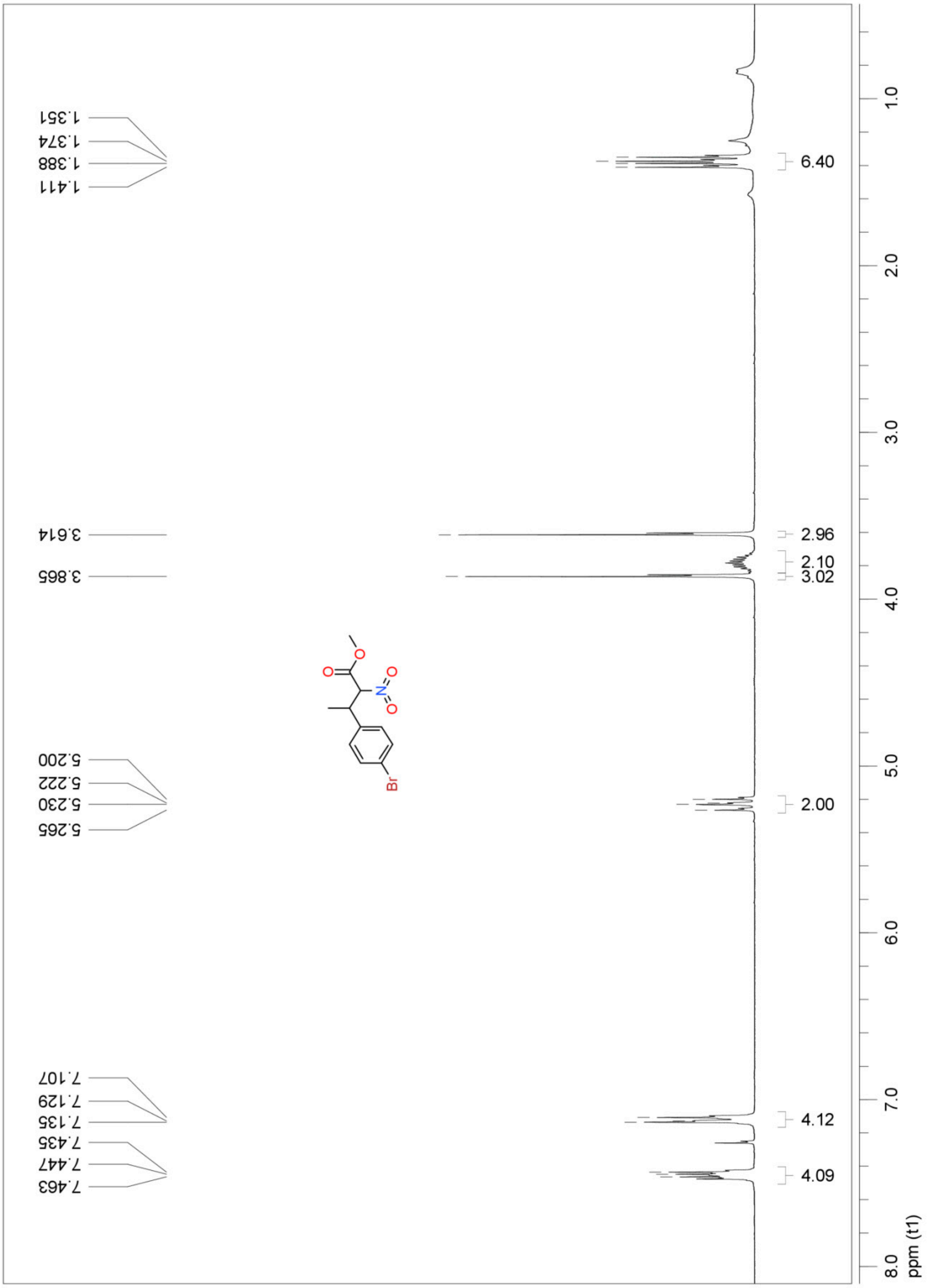
1.351
1.374
1.388
1.411
3.614
3.865
5.200
5.222
5.230
5.265
7.107
7.129
7.135
7.435
7.447
7.463
6.40
2.96
2.10
3.02
2.00
4.12
4.09
1.0
2.0
3.0
4.0
5.0
6.0
7.0
8.0
ppm (t1)
Br

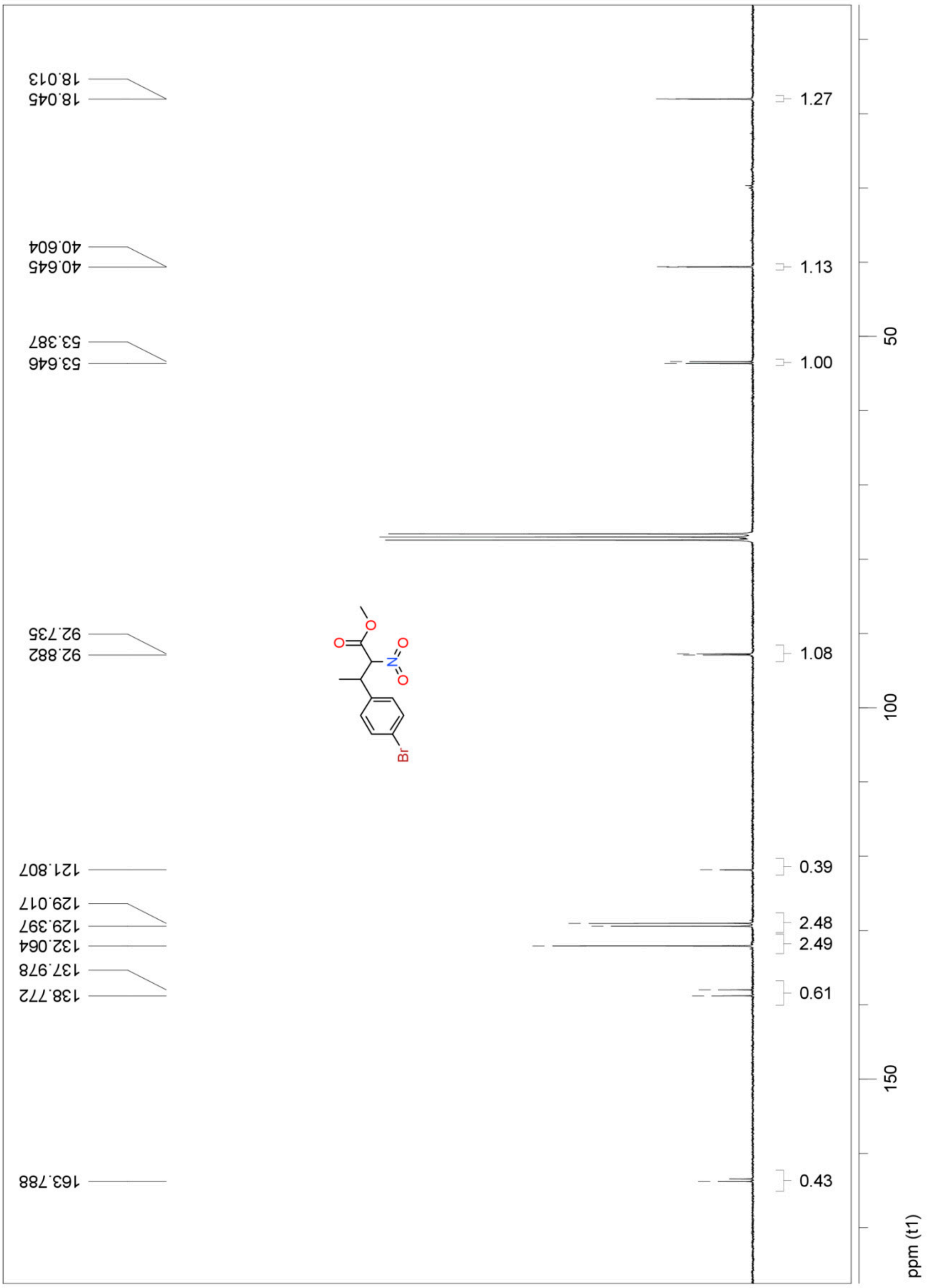
18.013
18.045
40.604
40.645
53.387
53.646
92.735
92.882
121.807
129.017
129.397
132.064
137.978
138.772
163.788
1.27
1.13
1.00
1.08
0.39
2.48
2.49
0.61
0.43
50
100
150
ppm (t1)
O
O
N
O
O
Br

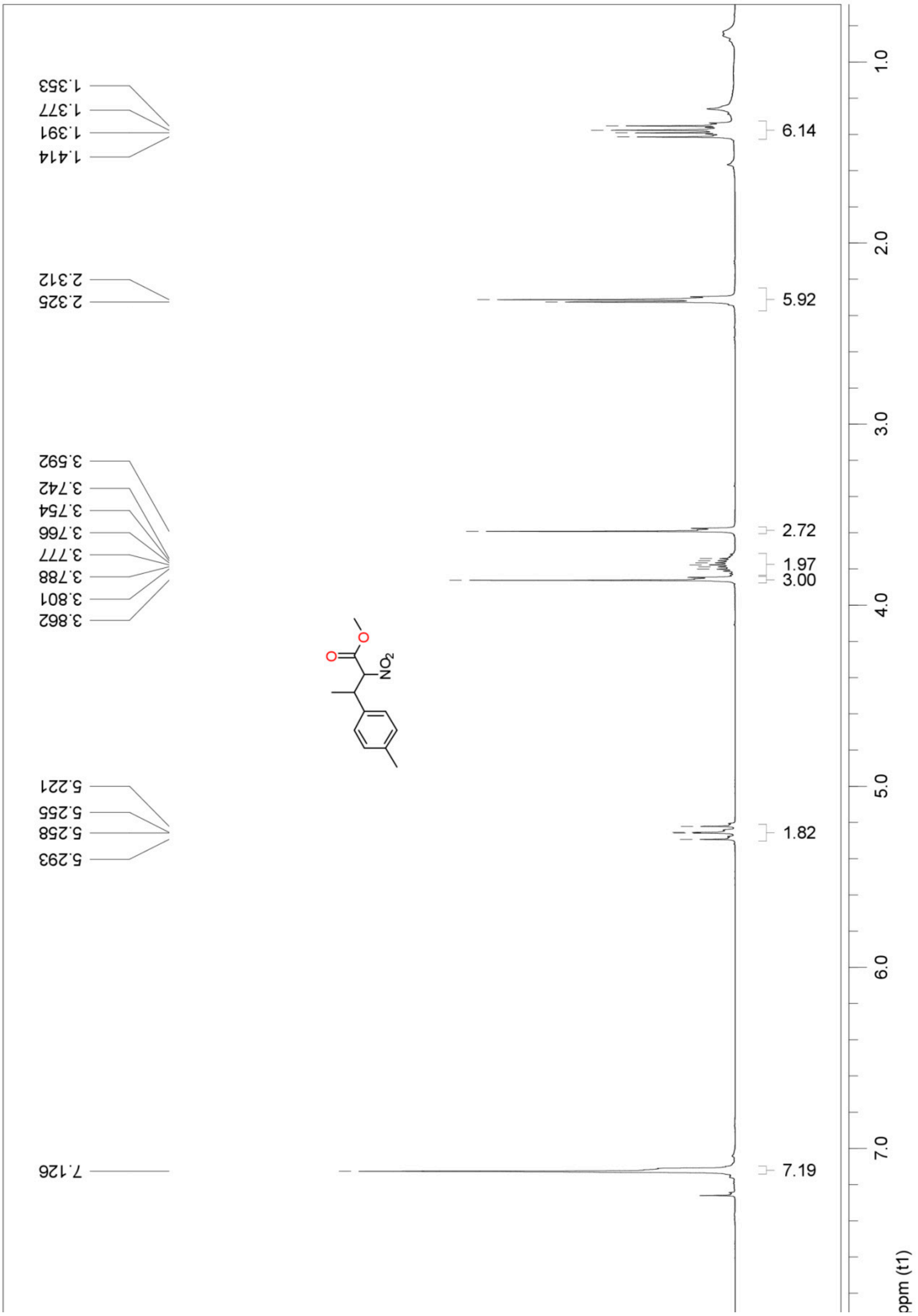
1.353
1.377
1.391
1.414
2.312
2.325
3.592
3.742
3.754
3.766
3.777
3.788
3.801
3.862
5.221
5.255
5.258
5.293
7.126
6.14
5.92
2.72
1.97
3.00
1.82
7.19
NO_2
1.0
2.0
3.0
4.0
5.0
6.0
7.0
ppm (t1)

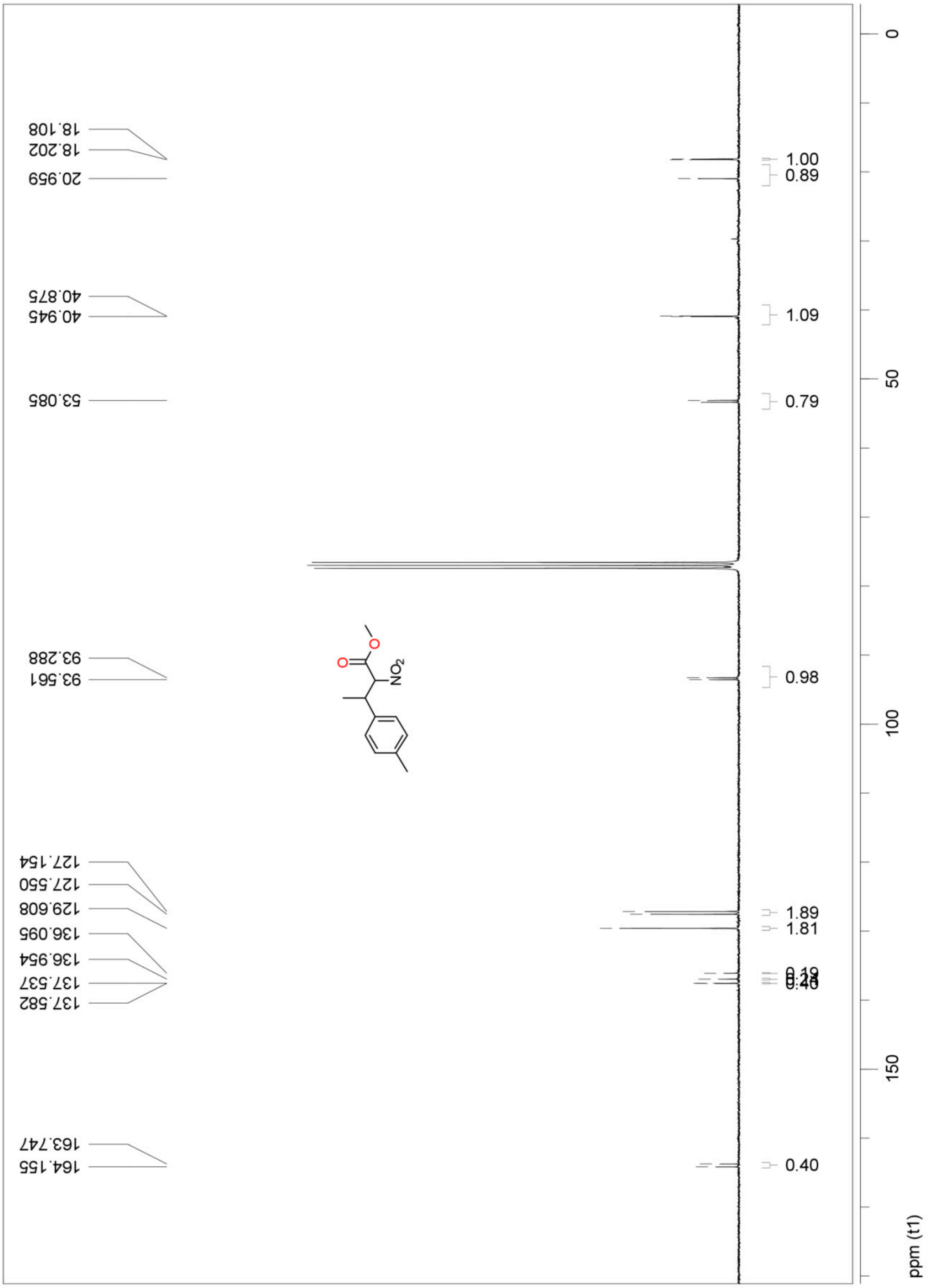
18.108
18.202
20.959
40.875
40.945
53.085
93.288
93.561
127.154
127.550
129.608
136.095
136.954
137.537
137.582
163.747
164.155
NO_2
1.00
0.89
1.09
0.79
0.98
1.89
1.81
0.40
0
50
100
150
ppm (t1)

2.4 *HPLC data of nitroalkanes 4*

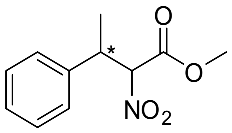

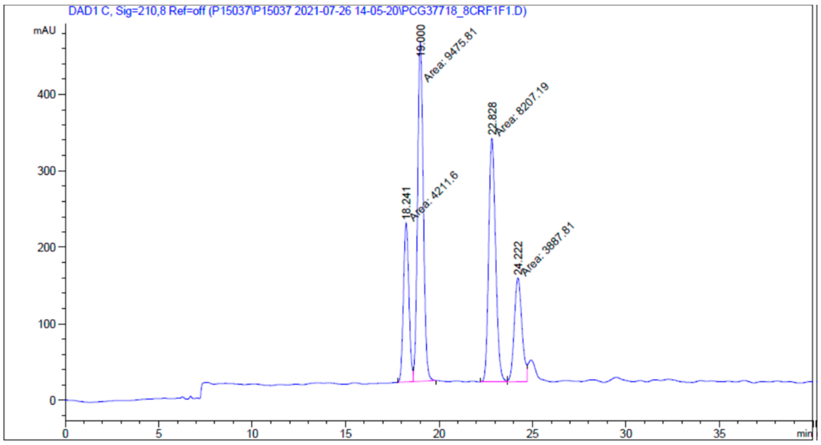

Peak #	RetTime [min]	Type	Width [min]	Area [mAU*s]	Height [mAU]	Area %
1	18.241	MF	0.3379	4211.59766	207.76465	16.3352
2	19.000	FM	0.3560	9475.81445	443.66376	36.7530
3	22.828	MF	0.4293	8207.19238	318.64307	31.8325
4	24.222	MF	0.4767	3887.80957	135.91457	15.0793

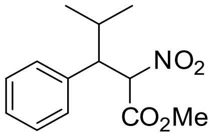

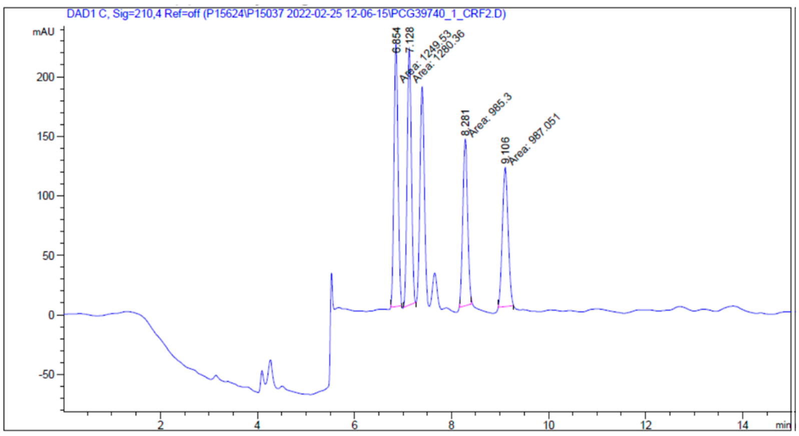

Peak #	RetTime [min]	Type	Width [min]	Area [mAU*s]	Height [mAU]	Area %
1	6.854	MM	0.0940	1249.53137	221.47246	27.7535
2	7.128	MM	0.0990	1280.36475	215.46544	28.4383
3	8.281	MM	0.1170	985.29993	140.30157	21.8846
4	9.106	MM	0.1403	987.05127	117.26270	21.9235

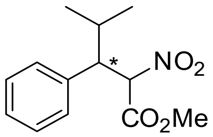

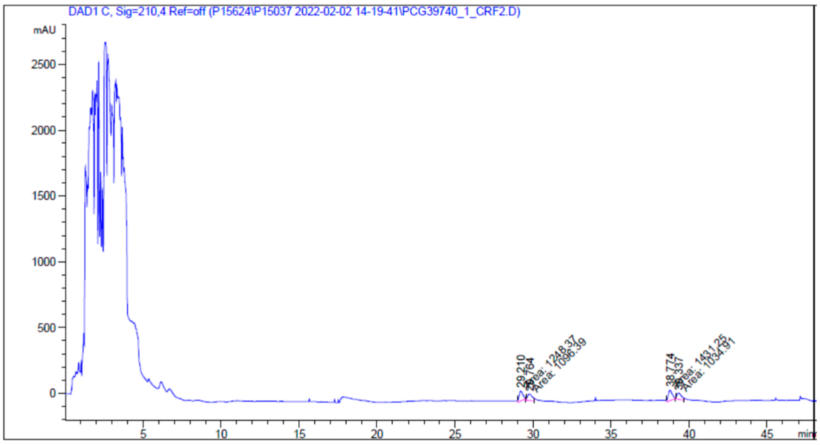

Peak #	RetTime [min]	Type	Width [min]	Area [mAU*s]	Height [mAU]	Area %
1	29.210	MM	0.2773	1248.37207	75.02459	25.9487
2	29.764	MM	0.3641	1096.39026	50.18269	22.7896
3	38.774	MM	0.2975	1431.24573	80.19376	29.7499
4	39.337	MM	0.3529	1034.91284	48.87428	21.5117

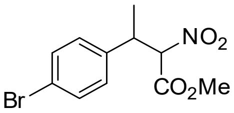

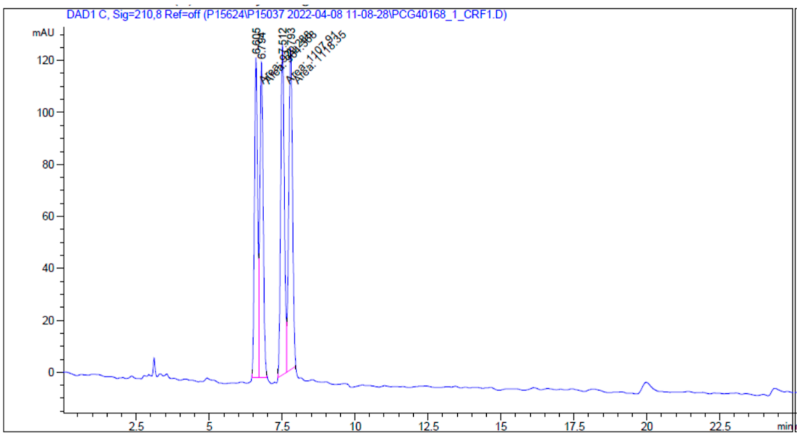

Peak #	RetTime [min]	Type	Width [min]	Area [mAU*s]	Height [mAU]	Area %
1	6.605	MF	0.1256	929.28839	123.30390	22.5560
2	6.794	FM	0.1324	964.36804	121.41569	23.4075
3	7.512	MF	0.1452	1107.90845	127.16387	26.8916
4	7.793	FM	0.1504	1118.34741	123.93054	27.1449

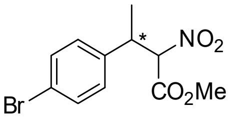

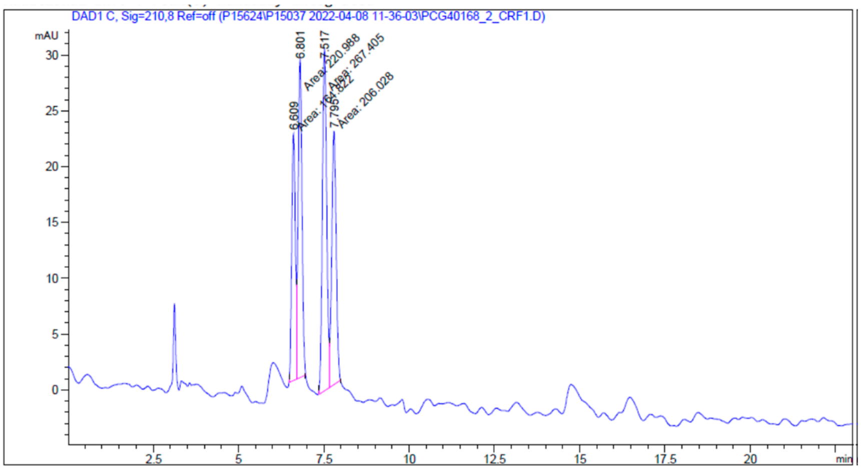

Peak #	RetTime [min]	Type	Width [min]	Area [mAU*s]	Height [mAU]	Area %
1	6.609	MF	0.1240	164.82224	22.14586	19.1823
2	6.801	FM	0.1286	220.98792	28.64508	25.7189
3	7.517	MF	0.1452	267.40518	30.69749	31.1210
4	7.795	FM	0.1510	206.02779	22.74287	23.9778

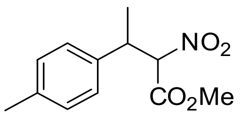

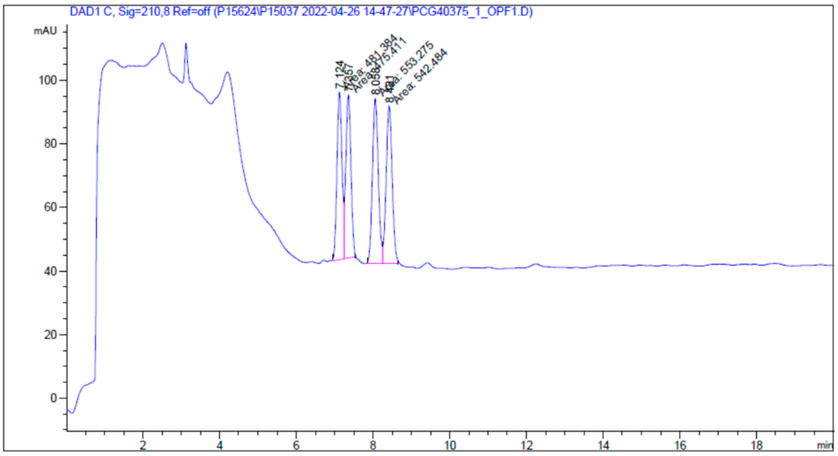

Peak #	RetTime [min]	Type	Width [min]	Area [mAU*s]	Height [mAU]	Area %
1	7.124	MF	0.1522	481.38358	52.72947	23.4529
2	7.357	FM	0.1543	475.41125	51.33826	23.1619
3	8.058	MF	0.1774	553.27496	51.98196	26.9554
4	8.421	FM	0.1826	542.48425	49.52488	26.4297

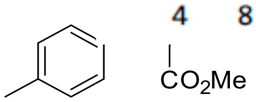

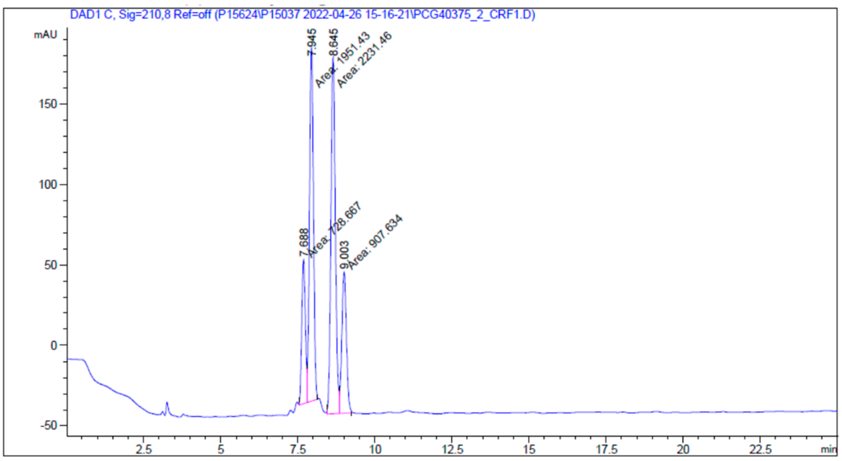

Peak #	RetTime [min]	Type	Width [min]	Area [mAU*s]	Height [mAU]	Area %
1	7.688	MF	0.1357	728.66669	89.46865	12.5218
2	7.945	FM	0.1475	1951.42749	220.53372	33.5343
3	8.645	MF	0.1679	2231.46240	221.48985	38.3466
4	9.003	FM	0.1724	907.63434	87.72820	15.5973

2.5 *Determination of the absolute configuration of nitroalkane 4a*

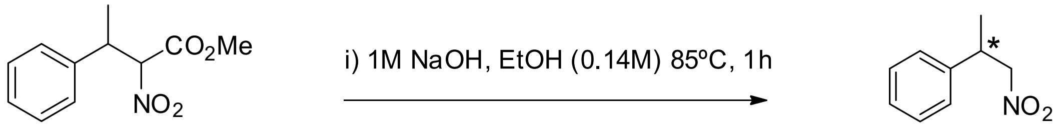

4a **8a**

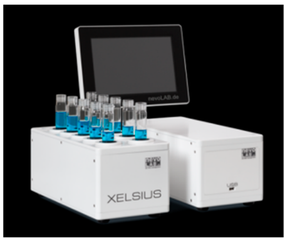

Figure 1. Xelsius apparatus

The reaction was performed using a Xelsius apparatus (Figure 1) based on data extracted from a literature procedure.[3] In a Xelsius vial, a solution of compound **4a** in Ethanol (0.14M) 1M solution of sodium hydroxide was dropwise added and the mixture was heated at 85ºC for 1h and, monitored by TLC after disappearance of starting material. Then, the mixture was allowed to warm to room temperature and the solvent was eliminated under reduced pressure. The remaining salts were dissolved in tetrahydrofuran (0.17 M respect compound **4a**) and an equal volume of 1M hydrochloric acid was added. The mixture was heated at 85ºC for 1h. After this time, the mixture was allowed to warm to room temperature and diluted with ethyl acetate. The aqueous layer was extracted with ethyl acetate. The combined organic layers were dried using Na_2SO_4, filtered and concentrated in vacuo. Compound **8a** was obtained as colorless oil in 27% yield.

^{1}HNMR ($CDCl_3$, 300 MHz) 1.35 (s, 3H) 1.37 (s, 3H) 3.57-3.65 (m, 2H) 4.43-4.57 (m, 4H) 7.19-7.33 (m, 10H)

GC-MS 4.15 min; m/z = 165.19

1. [3] A.E. Metz, M.C. Kozlowski *2-Aryl-2-nitroacetates as Central Precursors to Aryl Nitromethanes, α-Ketoesters, and α-Amino Acids J.Org.Chem* **2013**, *78*,2, 717-722

The optical rotation of compound **8a** was measured using a polarimeter obtaining an experimental value of $[\alpha]^{25}_{D} = +18$ (c = 0.5 in $CHCl_3$). Enantiomeric excess was measured using chiral HPLC column Phenomenex Cellulose 3_Hex_IPA_95:5_0.75 mL/min.

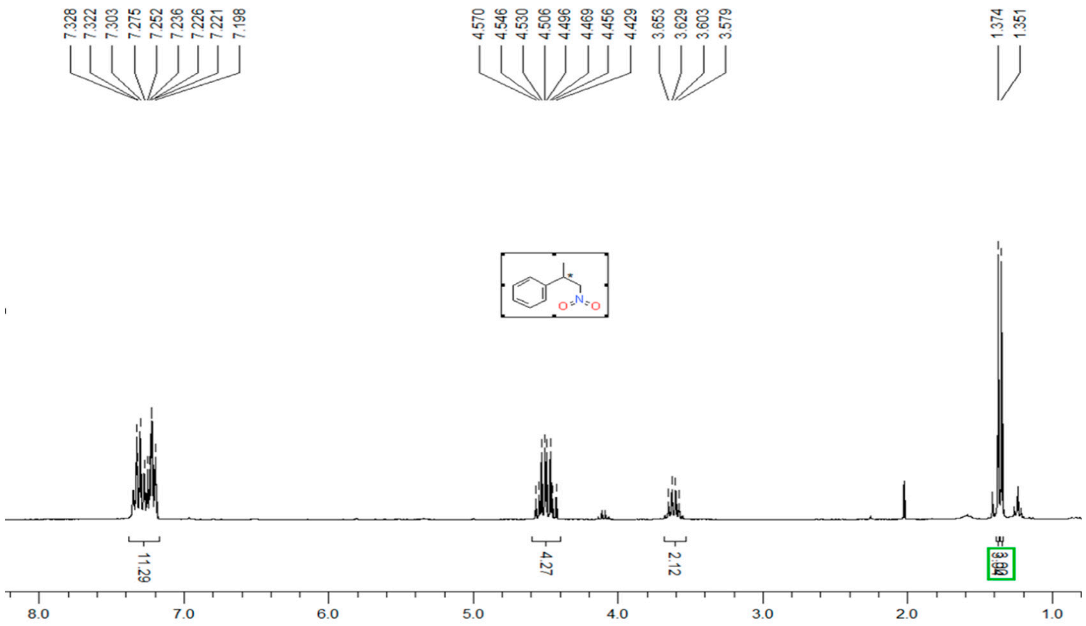

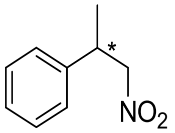

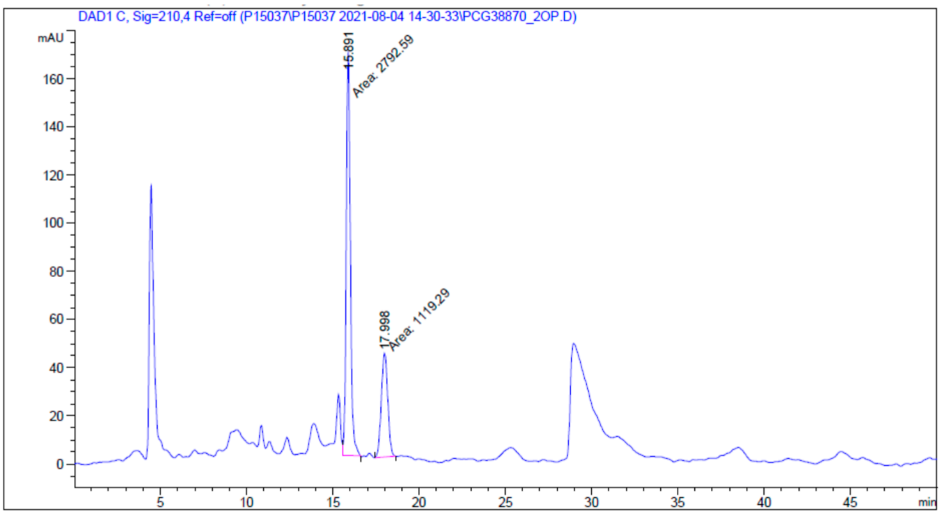

Peak #	RetTime [min]	Type	Width [min]	Area [mAU*s]	Height [mAU]	Area %
1	15.891	FM	0.2760	2792.58862	168.66377	71.3875
2	17.998	MM	0.4324	1119.28711	43.14579	28.6125
